# Guidelines for model adaptation: A study of the transferability of a general seagrass ecosystem Dynamic Bayesian Networks model

**DOI:** 10.1002/ece3.9172

**Published:** 2022-08-04

**Authors:** Paula Sobenko Hatum, Kathryn McMahon, Kerrie Mengersen, Paul Pao‐Yen Wu

**Affiliations:** ^1^ School of Mathematical Sciences, Science and Engineering Faculty Queensland University of Technology Brisbane Queensland Australia; ^2^ Centre for Marine Ecosystems Research, School of Science Edith Cowan University Joondalup Western Australia Australia

**Keywords:** complex systems, ecosystems management, model transfer, seagrass

## Abstract

In general, it is not feasible to collect enough empirical data to capture the entire range of processes that define a complex system, either intrinsically or when viewing the system from a different geographical or temporal perspective. In this context, an alternative approach is to consider model transferability, which is the act of translating a model built for one environment to another less well‐known situation. Model transferability and adaptability may be extremely beneficial—approaches that aid in the reuse and adaption of models, particularly for sites with limited data, would benefit from widespread model uptake. Besides the reduced effort required to develop a model, data collection can be simplified when transferring a model to a different application context. The research presented in this paper focused on a case study to identify and implement guidelines for model adaptation. Our study adapted a general Dynamic Bayesian Networks (DBN) of a seagrass ecosystem to a new location where nodes were similar, but the conditional probability tables varied. We focused on two species of seagrass (*Zostera noltei* and *Zostera marina*) located in Arcachon Bay, France. Expert knowledge was used to complement peer‐reviewed literature to identify which components needed adjustment including parameterization and quantification of the model and desired outcomes. We adopted both linguistic labels and scenario‐based elicitation to elicit from experts the conditional probabilities used to quantify the DBN. Following the proposed guidelines, the model structure of the general DBN was retained, but the conditional probability tables were adapted for nodes that characterized the growth dynamics in *Zostera* spp. population located in Arcachon Bay, as well as the seasonal variation on their reproduction. Particular attention was paid to the light variable as it is a crucial driver of growth and physiology for seagrasses. Our guidelines provide a way to adapt a general DBN to specific ecosystems to maximize model reuse and minimize re‐development effort. Especially important from a transferability perspective are guidelines for ecosystems with limited data, and how simulation and prior predictive approaches can be used in these contexts.

## Graphical abstract

The study in this paper focuses on a model transferability case study to design and implement model adaption guidelines. Our guidelines demonstrate how to adapt a general DBN to specific ecosystems in order to maximise model reuse and minimise re‐development effort, which is especially, which is especially relevant from a transferability perspective, are guidelines for ecosystems with limited data.

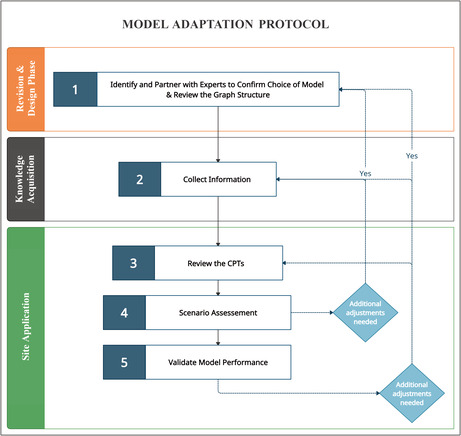

## 1 INTRODUCTION

Ecological models and related decision‐support frameworks support defining problems, conveying ecological concepts and relationships, characterizing potential system responses to management perturbations, and evaluating alternative management policies (McCann et al., 2006). High‐quality data are fundamental to such modeling, yet it may be expensive, insufficient, or indeed unavailable. Numerous ecological studies have shown the effect of limited data on the predictive accuracy of models (e.g., Chen, 2003; Pearce & Boyce, 2006; Vaughan & Ormerod, 2003). One approach to provide modeling decision support in challenging data environments is to transfer a general model from another context to the one being managed.

The difficulty of collecting enough empirical data to capture the entire range of processes that define a complex system is exacerbated, when viewing the system from a different geographical or temporal perspective (Clark, 2001; Yates et al., 2018). When a general model is developed to illustrate the “ecological causal web” of key variables and their influences on an ecosystem (Marcot et al., 2006), it becomes potentially applicable to a wide range of domains. As long as the core character of the decision‐making process remains the same, model transfer can be more cost effective and enhance model uptake.

In ecology, there are many examples of spatial and temporal transfer of species distribution models (Bridge et al., 2020; Sequeira et al., 2016, 2018). Models have also been used in different locations (Barbosa et al., 2009; Lauria et al., 2015; Randin et al., 2006) and times (Barbosa et al., 2009; Moreno‐Amat et al., 2015; Rapacciuolo et al., 2012; Tuanmu et al., 2011) to which they were originally developed. However, there remain many challenges in model transferability. These include challenges with the theory (Yates et al., 2018), data use (Aubry et al., 2017; Morán‐Ordóñez et al., 2017), methods for transfer for different modeling methods (Heikkinen et al., 2012; Sequeira et al., 2018), and resultant interpretation of transferability (Wenger & Olden, 2012).

Ongoing efforts have been made to better understand the patterns and determinants of model adaptation and transferability. For example, Lauria et al. (2015) examined and evaluated model transferability across regions using a four‐step framework: model selection, model evaluation, model transferability between regions, and model mapping. In the methodology developed by Lauria et al. (2015), the smallest Akaike Information Criterion was used to determine the best model; Spearman rank and the coefficient of determination (*R* ^2^) were used to evaluate the relationship between observed and predicted values, and at the “mapping” stage, species‐environment relationships were used to interpolate the distribution of the species in the same geographic area in which the model was calibrated. Moon et al. (2017) created a more analytical approach to characterize a given model's application niche by synthesizing information from databases, previous research, and models in unique and innovative applications to produce performance curves indicating whether a specific model is acceptable or not for a distinct context.

Undeniably, correlative models relating ecological metrics to environmental and spatial predictors are often used and play a critical role in supporting management and conservation efforts worldwide. However, such models consider only a subset of the relevant ecological processes. Even when other variables are included in models, there may be no data available for that variable nor an understanding of how it interacts with other variables. This makes predictions of ecological interactions beyond the range of observed values very challenging. Dealing with ecological problems is inherently complex since ecosystems are composed of heterogeneous, complex networks with nonlinear relationships and limited predictability (Folke et al., 2004; Starfield, 1997). This is due to multiple interactions that occur within ecosystems and between system components across temporal and spatial dimensions (Green et al., 2005).

In this paper, we consider the challenge of adapting a Dynamic Bayesian Networks (DBN) model of an ecosystem developed in a generic context and transferring it to a specific context. DBNs have been widely used as a tool to assist in ecological research and management in numerous studies where network structures capture nonlinear, dynamic processes in response to natural and anthropogenic stressors (Maxwell et al., 2015; Trifonova et al., 2015; Wu et al., 2017). They can integrate disparate and often limited data and capture uncertainties and complexities inherent in natural systems (Marcot & Penman, 2019). DBNs are temporal extensions of Bayesian networks (BN), which are probabilistic graphical models that use a set of nodes (variables of interest) to represent a system. Nodes can be deterministic or stochastic, with the later represented by continuous probability distributions. The structure of a BN is defined graphically, where each variable within the DBN network is presented as a node with directed links forming arcs that express hypothesized causal or directed associative relationships conditional probabilities tables (CPTs; Koski & Noble, 2011).

Marcot et al. (2006) describe guidelines for developing and updating BNs in the context of ecological assessment, with steps to create, test, calibrate, and update BN models at three levels: alpha, beta, and gamma. The alpha‐level BN models are developed by building influence diagrams depicting the hypothesized “causal web” of key elements that impact a species or ecological outcome of interest. The beta‐level model is produced following a formal revision of the model structure and CPT values by species experts who were not engaged in the development of the model. The gamma‐level or final application model is created by further testing, calibrating, validating and updating the beta‐level model.

However, to our knowledge, context‐specific guidelines and examples for transferring an ecological DBN have not yet been explored in the literature (Table S2). The ecological context of interest in this paper is seagrass. Seagrass ecosystems are widely recognized as crucial ecosystems in the coastal zone, with essential functions contributing to multiple marine ecosystems (Hemminga & Duarte, 2000; Pachauri et al., 2014). As plants living in shallow coastal waters, seagrass are typically subjected to anthropogenic stressors, such as water quality degradation and coastal development (Cambridge & McComb, 1984; Orth et al., 2006). Consequently, understanding the risks posed to these systems and how they respond to successive disturbances is essential for improved management (McCann et al., 2006).

Wu et al. (2018) developed a DBN model to predict seagrass meadow resilience to dredging disturbances. The model focuses on three genera and locations: *Amphibolis* in Jurien Bay, *Halophila* in Hay Point, and *Zostera* in Pelican Banks, Gladstone. Overall system interactions were evaluated, such as light loss due to dredging (the hazard), as well as ecosystem characteristics such as lifehistory characteristics exhibited by genera and local environmental variables. The general DBN model was also used to predict how dredging affects the resilience of seagrasses from 28 locations throughout the globe (Wu et al., 2017). However, species‐specific modifications and the overall model's applicability to particular areas have yet to be studied. Therefore, we attempted to assess the model transferability from global to local scale and from genera to seagrass species.

In the following, we use the terms model adaptation and model transfer interchangeably. We consider a structured approach for the model adaptation. General guidelines are introduced to adapt an existing DBN to a new context and validate the new model with limited data. The proposed guidelines and lessons acquired from this research may also be extended to other contexts and serve as a guide for the reuse and modification of different models, particularly for locations with limited data.

## 2 MATERIALS AND METHODS

### 2.1 Overview of the guidelines

**FIGURE 1:**
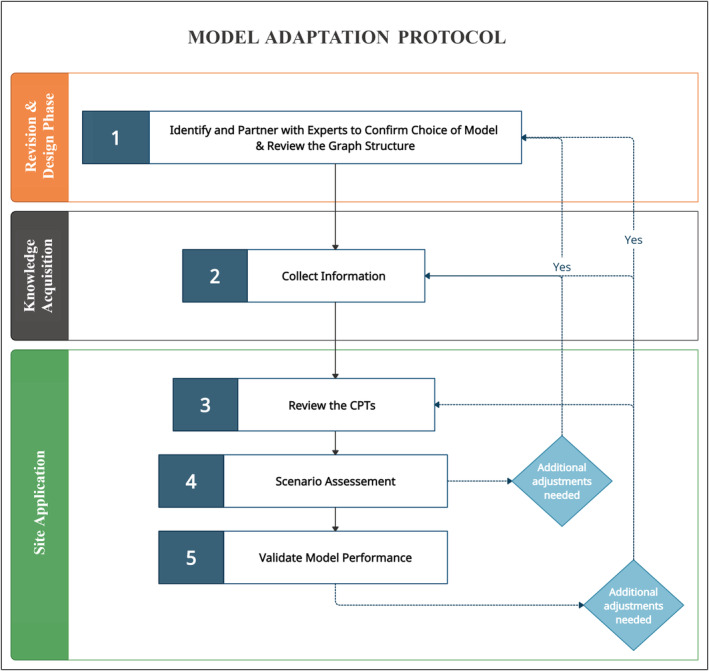
Stepwise methodology flowchart for adapting an existing model using information from data, literature, and expert knowledge

Our proposed guidelines have three main stages: revision and design phase, knowledge acquisition, and site application (Figure 1). The first phase encompasses a single step (Step 1) in which the following tasks are performed: Identifying and collaborating with experts to assess the transferability of the chosen model. Thus, once an agreement has been reached, the structure of the model is revised. For example, how nodes are linked and which states should be assigned to each node. Key elements in the functioning of the environmental system are also identified in this stage. The second stage focuses on identifying available information for the study (Step 2). This may include experimental or observational data, models published, gray literature, and expert knowledge for the study area. The third stage is subdivided into three steps (Steps 3, 4, and 5) that are iterated through until an appropriate local model is obtained, given the available information. In the first part, information acquired in stage 2 is used to update the CPTs in the model (Step 3). The second part involves a general evaluation of the model through sensitivity and scenario assessment (Step 4). Finally, the proposed model is evaluated against observed data when possible and appropriate (Step 5).

**FIGURE 2:**
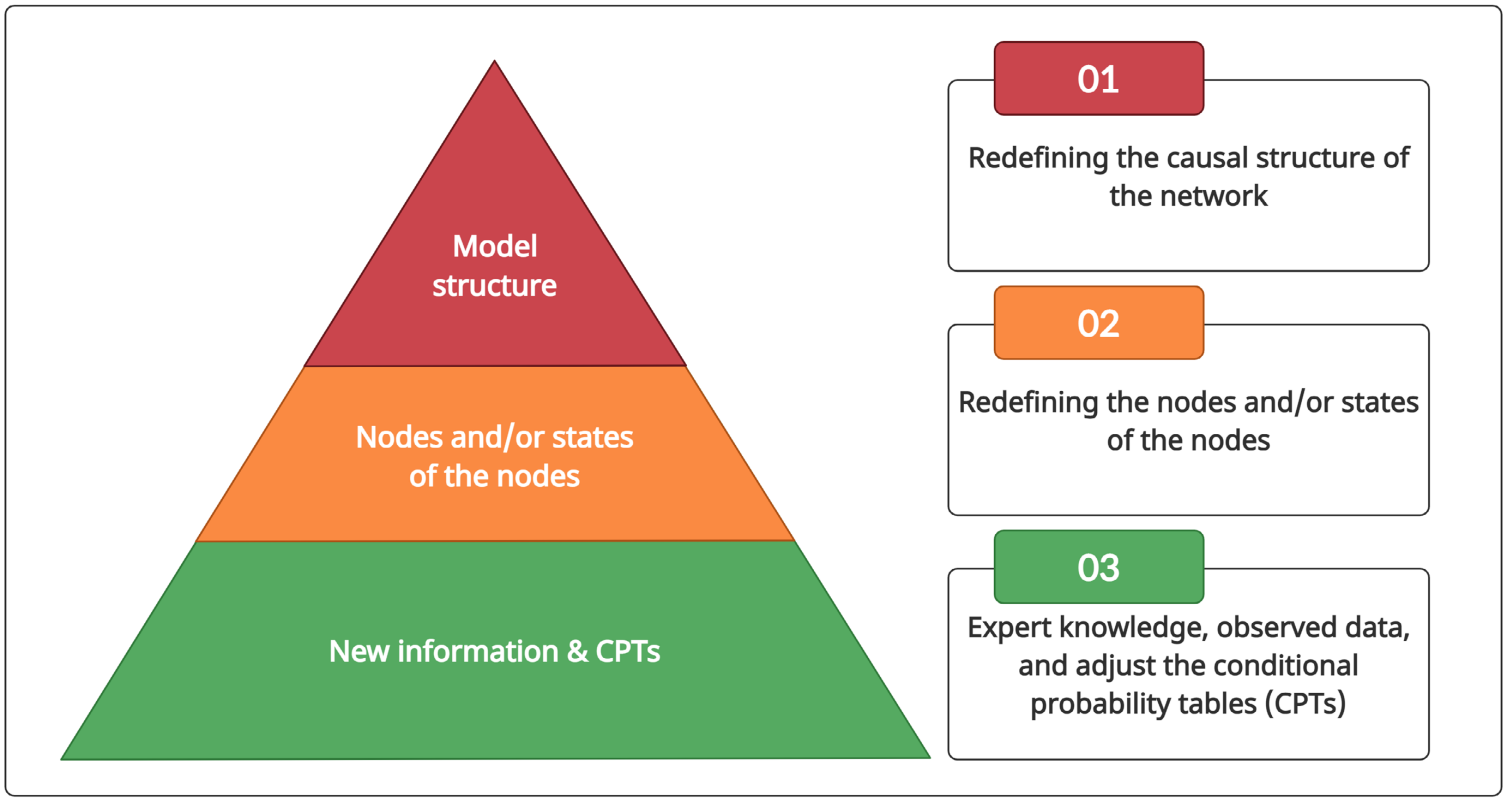
Order of preference for components to be adjusted in a DBN model. The pyramid suggests that it is most preferable to update only the CPTs, while the pointed top indicates it is least preferable to change the model structure.

Note that the steps shown in Figure 1 can be performed in a different order depending on the context. for example, step 2 may occur before or concurrently with step 1. Furthermore, it is essential to note that, unless there is a significant change in system processes, it is typically recommended to preserve as much of the model structure as possible (Grzegorczyk & Husmeier, 2009). When adapting a DBN model to a new context, it is thus most preferable to change the CPTs, redefine the node(s) and/or change the states of the node(s) rather than redesign the model structure (Figure 2).

#### 2.1.1 Step 1: Identify and partner with experts to confirm choice of model and review the graph structure

The task of the experts in this step is to use their own knowledge and other relevant information, such as peer‐reviewed literature, to confirm the choice of a DBN for the problem at hand and to review the model structure (Koski & Noble, 2011). The review procedure should consider the model's objectives, choice of relevant variables and response(s) (nodes), causal links (directed arrows), spatial and temporal scales, and other key elements. The model should be detailed enough to reflect the relevant ecological structure and processes of the system under consideration.

The research team has three main tasks to facilitate this step (Figure S1). The first task is to identify experts with relevant knowledge. Where possible, it is helpful to convene a pool of experts with both overlapping and complementary expertise about the system of interest (Drescher et al., 2013; Martin et al., 2012). Knol et al. (2010) and Caley et al. (2014) provide a variety of formal processes that can be used to identify experts.

The second task is to define the elicitation tool. An important design choice is between individual or group elicitation of information from experts. Individual elicitations may allow for more targeted questioning, explanation and feedback (Knol et al., 2010; Page et al., 2012), while groups may produce better predictions than individual assessments in certain situations (Surowiecki, 2004). When using multiple experts, separate opinions may be sought and aggregated, or group agreement may be pursued (Martin et al., 2012). Elicitation may take place in various forms, including via interviews, questionnaires or the use of specialized software (Cooke, 1994; O'Leary et al., 2009; Steinert, 2009). An alternative group approach is the Delphi method, in which experts provide information separately, then discuss the collective results and reconsider their responses in light of the responses of others. This approach has been widely used in ecology (Delbecq et al., 1975; Kuhnert et al., 2010; MacMillan & Marshall, 2006).

The second task also involves designing the elicitation questions. The first question is to confirm that a DBN model is indeed appropriate for the problem at hand, and that the particular DBN model from the source location is appropriate to consider transferring to the target location. The subsequent questions will focus on what adjustments are required in the DBN nodes and directed links, as well as the number and definition of the states of the nodes. An important parallel consideration is what information may be available to inform the corresponding changes in the CPTs (to support step 3). This may be sourced from observation data, literature or experts. Examples of questions regarding the model structure are given in Figure S1. It should be kept in mind that the DBN should maintain a balance between detail and robustness, in that it should include sufficient detail to adequately describe the target system while ensuring that the DBN can be adequately quantified.

The third task is to carry out the elicitation procedure. A vital part of this process is the preparation of the experts. The experts should be advised in advance about the aim of the elicitation and the elicitation tool. In many cases, it is helpful to provide background reading to ensure a common baseline understanding, as well as some training in probability to ensure appropriate calibration of responses. See Burgman et al. (2006) for a comprehensive review of techniques for eliciting expert judgment.

#### 2.1.2 Step 2: Collect information

Given confirmation of the suitability of the DBN and the model's structure, the next step is to collect information required to quantify the CPTs underpinning nodes in the model. This involves determining what experimental or observational data are available, collecting it to fill the gaps, assessing the degree of understanding of key elements of the system, identifying information sources such as literature, model outputs, and expert knowledge.

A significant strength of a DBN is its capacity to combine diverse data sources in a dynamic, whole‐of‐system model (Caley et al., 2014; Uusitalo, 2007).The relative merit of these different sources has been well argued in the literature, with some authors preferring sitespecific empirical relations and site‐specific data (e.g., Cain, 2001; Tari, 1996), others advocating the advantages of expert knowledge (e.g., Smith et al., 2007) and some proposing both (Pollino et al., 2007). In the latter paper, the authors suggested using expert elicitation to offer a first estimate of the probability and observed data to revise this estimate.

It is common for Step 2 to occur concurrently with Step 1 and Step 3 since these steps are closely connected. Therefore, depending on the availability of information, adjustments to the model structure may be required at this point, thus requiring returning to step 1 (Figure 1).

#### 2.1.3 Step 3: Review the CPTs

After reviewing the model's structure, the CPTs for those nodes indicated by experts as requiring adjustments must be assessed and, modified using the information acquired in Step 2. A CPT underlies every node in a DBN, in which the data (expressed as probabilities) used to fill the CPTs must describe how a node changes in response to changes in its parents. As the DBN is a network, the effect of changing any variable is transmitted right through the network in congruence with the relationships expressed by the CPTs. The dynamic component allows the model to capture these interactions between variables and changes over time (Friedman et al., 2013).

The CPTs can be completed directly by the research team in conjunction with the experts if applicable or calculated using algorithms chosen based on the available data. Several methods are used to update CPTs, such as the Lauritzen–Spiegelhalter algorithm (a basic representation of Bayes'theorem), Gibbs sampling, Expectation Maximization (EM) or Gradient Descent, which are built into most BN software (Chen & Pollino, 2012). A popular choice is the EM algorithm, which can estimate conditional probabilities from data with missing values (Uusitalo, 2007; Watanabe & Yamaguchi, 2003). The algorithm works by iteratively imputing missing data (expectation) and estimating CPT values (maximization), terminating when a local maximum is found.

#### 2.1.4 Step 4: Scenario assessment

This stage aims to examine the behavior of the DBN model to ensure that the key variables and their connections are accurately represented. The particular focus is on whether adjusted CPTs or revised structural changes in the DBN behave as anticipated with respect to inferred probabilistic outcomes. This stage is particularly important for studies with limited or scant data. A popular approach is scenario assessment, a form of evaluation in which a range of plausible scenarios are assessed against the model's aims and objectives and compared to one another. The scenarios allow the DBN to be evaluated in the studied ecosystem given certain biological, environmental, and ecological conditions. Expertise, existing literature, and analogous studies may be used to build the scenarios and both the research team and experts should be involved in evaluating the results.

This stage may also involve a global sensitivity analysis to evaluate the network's response to the different scenarios employed. A sensitivity analysis may help determine which variables and states of variables influence the outcome, highlighting priority risks or important knowledge gaps (Pollino et al., 2007).

Based on the findings of these assessments; Steps 1, 2, and 3 should be critically reviewed to assess each component of the model, including the overall structure, node discretization, and CPT quantification. If the model shows unrealistic behavior, the research team in collaboration with the experts should consider modifying the CPTs, by either combining, splitting, or redefining the nodes and/or states of the nodes or readjusting the overall structure of the model until it provides a reasonable response (Marcot, 2012). The approach applied in this step will return a mutually agreed model.

#### 2.1.5 Step 5: Validate model performance

This step builds on the previous step by evaluating in more detail the agreed DBN after scenario assessment. There are a variety of model validation approaches; selecting the most appropriate one will rely on available data and the modeling objective. Where possible, data‐based validation is preferred, although if data are sparse then a qualitative evaluation of model outputs using experts may be used to validate model predictions (Chen & Pollino, 2012).

For the former case of data‐based validation, when sufficient data are available, cross‐validation is preferable where the data set is randomly split into training (building the DBN) and testing sets (validating the DBN). The model outputs are compared to the test data and evaluated using a metric such as logarithmic probability; root mean squared error, or prediction accuracy (Aguilera et al., 2011). If data are insufficient to do this, goodness‐of‐fit measures can be used as the same data is used to train and test the DBN. In either case, predicted state probabilities are compared to the observed state probabilities obtained from the data. Finally, if there are little or no data, qualitative evaluation procedures can be applied, such as using expert knowledge (Chen & Pollino, 2012). Here, an independent expert reviewer may verify whether the model's behavior is consistent with the current understanding of the system.

### 2.2 Guidelines in the context of the case study

In accordance with the general guidelines, the methodology used to adapt the model to our case study is presented below, broken down into the three stages depicted in (Figure 1) with the steps presented in detail.

#### 2.2.1 Arcachon Bay case study

Our case study includes two *Zostera* seagrass species located in Arcachon Bay, France: *Z. marina* and *Z. noltei*. Arcachon Bay is a tidal ecosystem, sheltering Europe's largest seagrass bed of dwarf grass (*Z. noltei*; Auby & Labourg, 1996). This species colonizes soft sandy to muddy sediments of shallow sheltered bays, often in intertidal areas. In the shallow subtidal sector around the channel edges, another species, *Z. marina* (eelgrass) grows forming smaller beds (Cognat et al., 2018). Seagrass mapping between 1989 and 2007 showed a severe decline of *Zostera* spp. from 2005, an estimated 33% reduction for *Z. noltei* (from 68.5 to 45.7 km^2^) and 74% (from 3.7 to 1.0 km^2^) for *Z. marina* meadows (Plus et al., 2010).

Although studies have suggested that factors such as climate change, eutrophication, increased geese grazing, wasting disease, herbicide contamination, or dredging activities may explain this decline, the exact reason for the loss of seagrass in Arcachon Bay is still unclear (Cognat et al., 2018; Plus et al., 2010). Therefore, transferring a whole‐of‐system DBN model, which integrates analysis of interactions and feedbacks across different components of the system to Arcachon Bay, provides a way to understand the ongoing seagrass dynamics and allow projections to support future decision making. Furthermore, such a model could be used to simulate and assess different management scenarios to support decision makers.

#### 2.2.2 Revision and design phase

##### Step 1: Identify and partner with experts to confirm choice of model and review the graph structure

**FIGURE 3:**
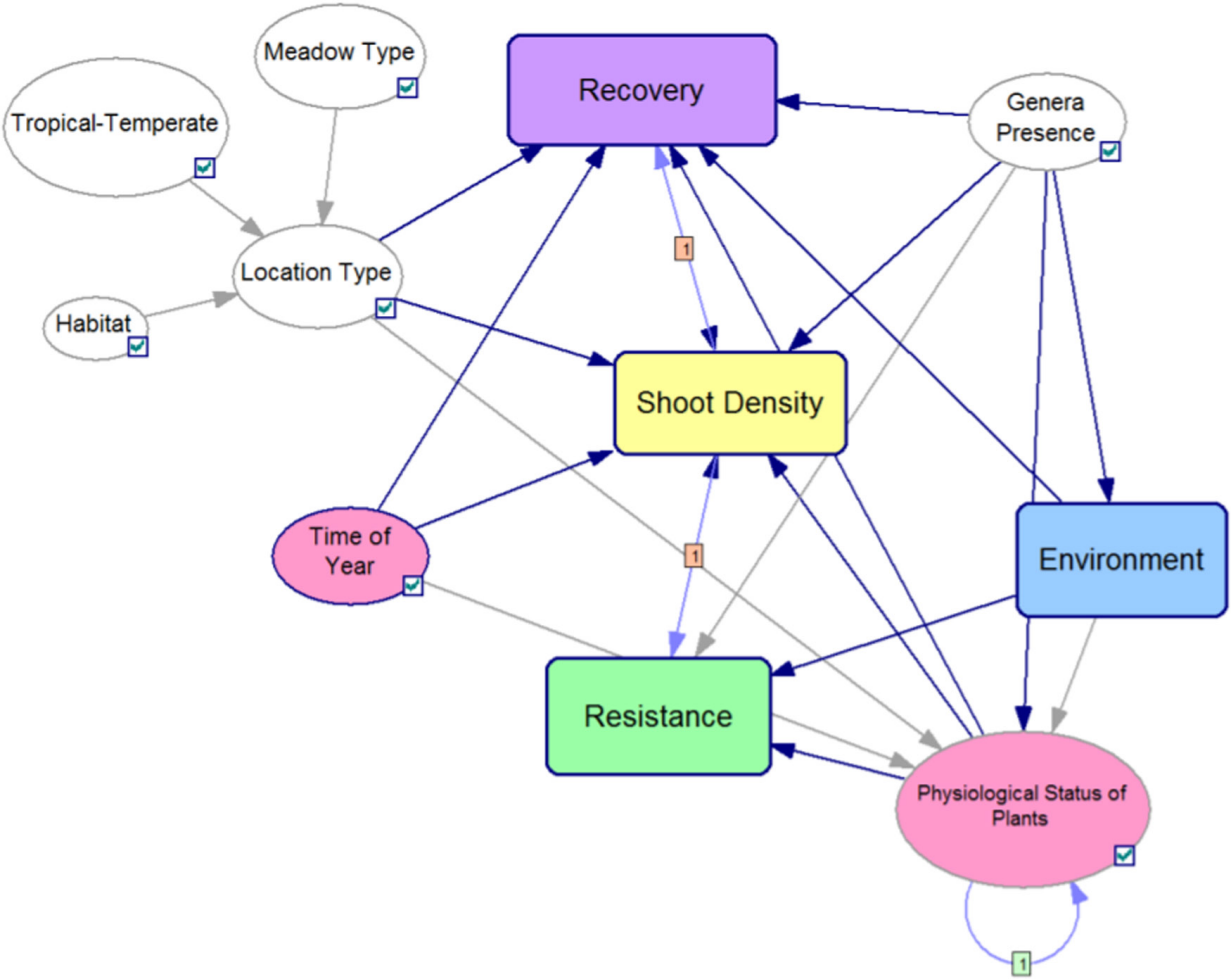
The overall DBN structure. Nodes are ovals and arrows denote causal parent–child relationships in the direction of the arc where a parent node (e.g., meadow type) influences a child node (e.g., location type); conversely, an absence of a link implies conditional independence. Rounded rectangles denote subnetworks. Nodes are colored as follows: White for site condition nodes, purple for recovery nodes, green for resistance nodes, blue for environmental nodes, yellow for population (shoot density) nodes, and pink for all other nodes. From “Timing anthropogenic stressors to mitigate their impact on marine ecosystem resilience Supplementary Information” by Wu et al. (2017), nature communications 8:1263, Figure S7.

The general DBN that was adapted in this study has a network structure comprised of 34 nodes organized into four themes, resistance (e.g., physiology), recovery (e.g., growth), site conditions (e.g., genera present), and environmental factors (e.g., light; Figures 3 and S2a,b). The current framework uses hybrid and dynamic BNs containing discrete variables over multiple time stages. The temporal frequency of this DBN model is monthly time steps and the spatial extend is at the local level of the seagrass meadow.

The existing model was designed in such a way that it could be expanded to a wide range of ecological domains by capturing ecosystem dynamics and effects in a probabilistic risk framework. In transferring this model to Arcachon Bay, the modeling context was identified, in consultation with local experts, to center on the conservation of two particular seagrass species under the *Zostera* genera captured in the general model. They were sufficiently different that the conditional probabilities needed to be reviewed and adapted for factors relating to seasonal growth, reproductive cycle, baseline conditions and environmental input factors such as light.

A group of eight experts with good local knowledge of seagrass and marine ecology were identified and invited to participate in the evaluation of the merit of the proposed DBN for the target site (Table S1). Following a critical approval review of the DBN structure, experts were also invited to participate in the knowledge elicitation process. Local ecological knowledge, such as information regarding the target species' seasonal growth and reproduction dynamics, was therefore crucial for adapting the general DBN to the given ecosystem.

#### 2.2.3 Knowledge acquisition

##### Step 2: Collect information

The research team was responsible for guiding the experts through the tasks, encouraging discussion, and presenting results and analysis back to the experts. In addition, modelers worked collaboratively with domain experts in establishing relevant literature, data, and key biological and environmental processes that needed to be adapted for the case study. Communication with all experts was carried out entirely online, via Zoom and e‐mail, since face‐to‐face meetings were not feasible due to global pandemic travel restrictions.

In the first meeting with the experts, training was conducted for those unfamiliar with DBN and probabilistic quantification of CPTs. The case study was then presented with the overall aim to transfer the Wu et al. (2017) model to new sites in Arcachon Bay, focusing on two species of seagrasses (*Z. marina* and *Z. noltei*). Interviews were then held with the experts to assess the DBN nodes (and/or states) directed linkages and confirm that the particular DBN model was appropriate for transferring to the target location. Since most experts already had experience in the BN CPT elicitation process, we reviewed and quantified the CPTs in parallel (See details in Section 2.2.4, Step 3).

The empirical data used here was provided by IFREMER (the French Institute for Research and Sea Exploitation) collected from nine sampling sites distributed over the whole of the Bay selected for their different depths, environmental conditions, and seagrass density (Cognat et al., 2018). Although we have data for nine sites, only four sites, FONT, GAIL, ILE, and ROCH, were considered for tuning model parameters (light thresholds) and validation analysis because these sites were considered to be in good physiological condition and historically had not declined (Florian Ganthy, pers. comm.).

Seagrass shoot density, benthic light, and temperature data from a 1‐year field survey (December 2015–December 2016) were used to test and validate the model. For each site, measurements of shoot density were collected monthly, while light intensity (μmols m^−2^ s^−1^) and temperature (°C) were measured continuously at high frequency (10 min sampling rate). Unfortunately, no shoot density and biomass records were available for *Z. marina*, making it impracticable to validate the model for this species. To incorporate light data in DBN inference, we discretised light into states. The probability of light being in one of these states is based on simultaneous requirements of light intensity (mols m^−2^ day^−1^) and light duration per day (number of hours of saturation and compensation irradiance per day). Therefore, site‐specific information was required when establishing critical thresholds for water quality based on the responses of seagrass plants to light availability and minimum light levels. As this information was not available for our study area, we employed expert elicitation based on studies from similar sites in France and peer‐reviewed literature to estimate light thresholds and estimate baseline light patterns.

Like other plants, the light regime is the primary environmental factor influencing photosynthesis and the growth of seagrass (Dennison, 1987). The light required for growth and survival varies by species, location, and temperature (Kirk, 1994). The maximum photosynthetic rate which promotes plant growth occurs at saturating light conditions (above the light half‐saturation point *I*_k_). At lower light values, the compensation irradiance (*I*_c_) level captures when photosynthesis exactly balances respiration and primary metabolism is maintained but not growth. If light falls below *I*_c_, respiration is greater than photosynthesis, and there is not enough light for plant survival (Lee et al., 2007). In the existing DBN model, the probability of above or below saturation light is used to capture the optimal and suboptimal light conditions that support seagrass growth. Here, experts proposed to test two distinct ways to discretise the light factor to obtain evidence to support the use of a 2‐state (based only on *I*_k_) or a 3‐state (*I*_k_ and *I*_c_) light model. The thresholds used to discretise the light factor into those states are described below.

As light intensity thresholds were not well understood in our study area, we used a K‐nearest neighbors algorithm (k‐NN; Fix & Hodges, 1989) based on published data to apply to our area of study (See Supporting Information S1, and Table S3). For this approach, since photosynthetic parameters are related to temperature and show seasonal trends, we used the monthly temperature of Arcachon Bay to predict seasonal *I*_k_ and *I*_c_ thresholds. The saturation and compensation irradiance (*I*_k_ and *I*_c_, respectively) obtained from the k‐NN algorithm are summarized for *Z. noltei* in Table 1 (See Tables S3 and S4 for more information on *I*_k_ and *I*_c_, estimated for *Z. marina* and *Z. noltei* at the nine sampling sites). Both *I*_k_ and *I*_c_ were used to assess the number of hours of saturation and compensation light. From that, thresholds for light duration (*H*_sat_ and *H*_comp_) were required to determine the number of hours of saturation and compensation light per day was necessary to classify the daily light as above, below and/or below limitation state. Because this information was unknown for *Z. noltei* located at Arcachon Bay, we employed expert elicitation based on recorded data to set different combinations of *H*_sat_ and *H*_comp_ values (Table 2).

**TABLE 1:** Average monthly water temperature (temp, °C), saturation and compensation irradiance (*I*_k_ and *I*_c_, μmols photons m^−2^ s^−1^) estimated for *Z. noltei* located at FONT, GAIL, ILE and ROCH

	FONT			GAIL			ILE			ROCH		
	Temp	*I*_k_	*I*_c_	Temp	*I*_k_	*I*_c_	Temp	*I*_k_	*I*_c_	Temp	*I*_k_	*I*_c_
Jan	11	174	19	11	174	19	12	174	19	12	174	19
Feb	11	174	19	11	174	19	12	174	19	11	174	19
Mar	13	174	19	13	174	19	14	174	19	13	174	19
Apr	16	305	35	16	305	35	16	305	35	16	305	35
May	20	305	35	19	305	35	19	305	35	19	305	35
Jun	23	254	33	22	254	33	23	254	33	23	254	33
Jul	26	254	33	25	254	33	25	254	33	25	254	33
Aug	27	254	33	25	254	33	25	254	33	26	254	33
Sep	24	254	33	23	254	33	24	254	33	24	254	33
Oct	17	305	35	18	305	35	19	305	35	18	305	35
Nov	14	174	19	14	174	19	15	305	35	14	174	19
Dec	12	174	19	12	174	19	13	174	19	12	174	19

**TABLE 2:** The combination of the lengths of daily light periods thresholds (*H*_sat_ and *H*_comp_, hours) for *Z. noltei*

Model	Threshold ID	*H*_sat_	*H*_comp_
2‐state	Thdl‐1	4	–
	Thdl‐2	5	–
	Thdl‐3	5.5	–
	Thdl‐4	6	–
	Thdl‐5	7	–
	Thdl‐6	7.5	–
	Thdl‐7	8	–
	Thdl‐8	8.5	–
	Thdl‐9	9	–
3‐state	Thdl‐1	6	8.5
	Thdl‐2	6	9
	Thdl‐3	6	10
	Thdl‐4	6	11
	Thdl‐5	6	12
	Thdl‐6	7	8.5
	Thdl‐7	7	9
	Thdl‐8	7	10
	Thdl‐9	7	11
	Thdl‐10	7	12
	Thdl‐11	8	8.5
	Thdl‐12	8	9
	Thdl‐13	8	10
	Thdl‐14	8	11
	Thdl‐15	8	12
	Thdl‐16	8.5	8.5
	Thdl‐17	8.5	9
	Thdl‐18	8.5	10
	Thdl‐19	8.5	11
	Thdl‐20	8.5	12
	Thdl‐21	9	8.5
	Thdl‐22	9	9
	Thdl‐23	9	10
	Thdl‐24	9	11
	Thdl‐25	9	12

*Note*: The thresholds are separated for the 2‐state model.

After establishing the light intensity and duration thresholds, it was possible to estimate the number of days of light being in one of those states per month. The proportion of days of above saturation light in a month was represented by *δ*($x_{\text{abovesat}}^{\text{light}}$ *, t*), and the probability of above saturation light was encoded as *δ*($x_{\text{abovesat}}^{\text{light}}$ *, t*), *t* = {Jan, Feb,…, Dec}. The same equation was applied to model the probability of light being below saturation or below limitation. These probabilities were input as evidence to the DBN in simulating scenarios. Finally, we estimated the light conditions for all sites and used it as evidence for our model.

#### 2.2.4 Site application

##### Step 3: Review the CPTs

The process of reviewing the CPTs was undertaken with the expert team and took the form of scenarios, an intuitive way for experts to make sense of the evidence (Pennington & Hastie, 1993), and linguistic labels of certainty, extremely likely, very likely, likely, 50/50, unlikely, very unlikely, extremely unlikely and impossible. An iterative approach was adopted to maximize cognitive compatibility, as people find it challenging to think of probabilities with several conditioning factors to quantify the DBN (Uusitalo, 2007).

As stated above, based on expert agreement it was unnecessary to change the definition of nodes and the core model dynamics for our case study, so the overall structure of the DBN was retained. The focus was then on changes in the designation of probabilities and correspondents CPTs for these components that reflect the local system of interest (Step 3, Figure 1). The CPTs were used to capture the uncertainty and variation of multiple associations between species and their environment. To elicit the conditional probabilities for each node of interest from the experts, questions were phrased as follows “If seagrasses were under good conditions of light but show poor physiological status, what is the probability of the plants growing?”

**FIGURE 4:**
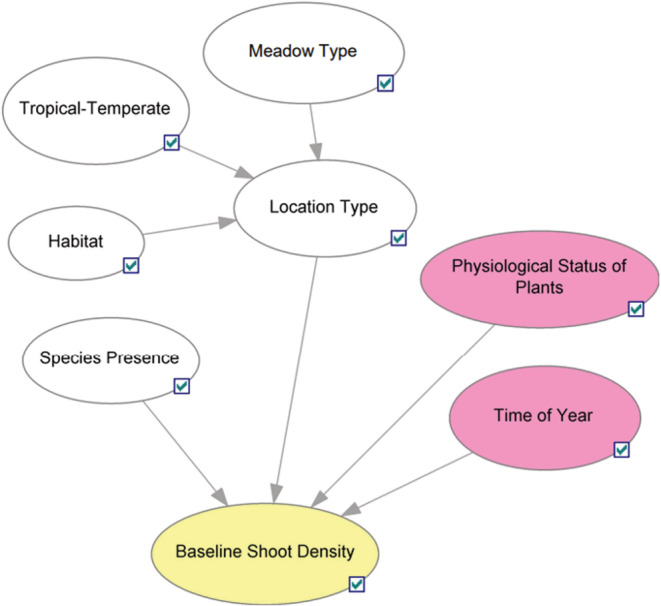
Simple model structure representing the relationship between a child node (baseline shoot density node) and all its parents (time of year, specie presence, location type, and physiological status of plants). Nodes are colored as follows: White for input nodes, yellow for population (shoot density) nodes, and pink for all other nodes.

Table 3 is an excerpt from expert‐elicited rules used to quantify the CPT of Baseline Shoot Density, and Figure 4 shows graphically the parent–child relationships between baseline shoot density and its parents (time of year, species presence, location type, and physiological status of plants). Note that this is just an excerpt from the full network, focusing on baseline shoot density. The baseline shoot density has four parent nodes. Each row in the table represents a unique scenario, which was formulated as questions that were posed to experts. Not applicable (NAs) are used to simplify notation by indicating when a scenario is independent of a given parent node.

**TABLE 3:** Illustration of a CPT calculation for the node baseline shoot density using expert elicitation

Antecedent	Antecedent	Antecedent	Antecedent	Baseline shoot density			
Time of year	Species presence	Location type	Physiological status of plants	High	Moderate	Low	Zero
ov, Dec, Jan, Feb	*Z. marina*	PTST	NA	Extremely Unlikely (0.01)	Extremely Unlikely (0.01)	50/50	50/50
Mar, Apr, May	*Z. marina*	PTST	NA	Unlikely (1/3)	Very Likely (5/6)	Very Unlikely (1/6)	Extremely Unlikely (0.01)
Jun, July	*Z. marina*	PTST	NA	Very Likely (5/6)	Very Unlikely (1/6)	Extremely Unlikely (0.01)	Extremely Unlikely (0.01)
Aug, Sept, Oct	*Z. marina*	PTST	NA	50/50	50/50	Extremely Unlikely (0.01)	Extremely Unlikely (0.01)
Nov, Dec, Jan, Feb	*Z. noltei*	PTIT	NA	Extremely Unlikely (0.01)	Unlikely (1/3)	Likely (2/3)	Extremely Unlikely (0.01)
Mar, Apr, May	*Z. noltei*	PTIT	NA	Unlikely (1/3)	Very Likely (5/6)	Very Unlikely (1/6)	Extremely Unlikely (0.01)
Jun, July	*Z. noltei*	PTIT	NA	Very Likely (5/6)	Very Unlikely (1/6)	Extremely Unlikely (0.01)	Extremely Unlikely (0.01)
Aug, Sept, Oct	*Z. noltei*	PTIT	NA	50/50	50/50	Extremely Unlikely (0.01)	Extremely Unlikely (0.01)

*Note*: The nodes time of year, specie presence, and location type (parent nodes) represent the causal factors of baseline shoot density node (child node). PTST represents persistent, temperate and subtidal, and PTIT represents persistent, temperate and intertidal.

During elicitations, we focused on updating the CPTs for nodes to capture the local growth dynamics of *Zostera* spp. meadows located in Arcachon Bay and seasonal variations in their population and life histories. Local knowledge of seagrass growth rates and reproductive success was required to express and calculate the relationships between nodes related to the main drivers of the fitness of seagrass. Temporal variations of growth rates and sexual reproduction (e.g., flowering shoots, seed production, and seed quality and density) between species and location were considered when updating the relevant conditional probability tables so that the interactions nodes and interactions between nodes captured the local conditions.

##### Step 4: Scenario assessment

The behavior of the structure was tested by the application of two light models, in which different numbers of states for the light node were used. Furthermore, for each light model, combinations of light thresholds were also considered to assess the posterior marginal probabilities for the shoot density node. Specifically, we were interested in a key outcome node which was shoot density and its change over time. Thus, it is possible to verify if the predictions obtained from the model are consistent with the current understanding of the system (Bogaert & Fasbender, 2007; Chen & Pollino, 2012; Uusitalo, 2007). Therefore, we simulated different light threshold scenarios for both 2‐ and 3‐state light formulations, and validated model predicted shoot density against observed shoot density. The simulations were conducted for each of the four sites in the Bay. The system response can be subdivided into two periods, the initialization period to establish the baseline pattern and the response period. A weighted mean approach was used as a comparative method in which multiple state probability trajectories are aggregated into a single trajectory. The weighted mean follows the approach of Wu et al. (2017).

##### Step 5: Validate model performance

The MSE was used as a distance metric to compute distances between simulated posterior marginal distribution for shoot density (probabilities for high, moderate, low and zero shoot density) against observed distributions of shoot density. Shoot density data collected in Arcachon Bay (Cognat et al., 2018) were used to validate the prediction of the model (See Table S4). We used a hierarchical ordinal regression analysis to transform the observed data into state probabilities of high, moderate, low and zero shoot density as follows:

$$g\left(y_{i},t\right)=\beta_{0,i}+\beta_{1,i}\;\sin\left(t|6\pi \right)+\beta_{2.i,\text{Site}}$$

Here, we use a Generalized Linear Mixed Model (GLMM), and *g* ^−1^(*y*_*i,t*_) represents the probability of state *i* (high, moderate, low and zero) of shoot density at time *t* (month of year). The regression has coefficients *β*_0_ and *β*_1_, which are the global intercept and the slope for the seasonal effect from months t, respectively, and coefficient *β*_2_, which is the random effect used to capture the differences between sites. The model was formulated with the Bayesian framework (Wu et al., 2015) and fitted with Hamiltonian Monte Carlo using the R package brms (Bürkner, 2018) using default, flat priors (i.e., uninformed priors).

## 3 RESULTS

### 3.1 Application of guidelines to case study

In this section, the results from the application of the guidelines for adapting a model to a case study are outlined. The results are broken down into three stages that include sub‐elements that can be viewed as a step‐by‐step process.

#### 3.1.1 Revision and design phase

##### Step 1: Identify and partner with experts to confirm choice of model and review the graph structure

Given the importance of local ecological knowledge, we obtained the participation of eight experts in seagrass and marine ecology. Among them, six experts came from the Ifremer, France, one from Edith Cowan University, Australia, and one from James Cook University, Australia. The panel of experts confirmed that the model could be transferred to the target location.

It was agreed that the model did not capture differences between species at local scales. Therefore, adjustments on factors used to capture the general health and growth of the two *Zostera* spp. were needed; these are summarized in Table 4. For example, although both species are perennial (persistent) in the Bay, *Zostera* beds display significant seasonal variations in density and biomass (Auby & Labourg, 1996). Tolerance and ability to acclimate to different environmental conditions, such as turbidity, salinity regimes and light availability, is also known to vary between species (Cognat et al., 2018; Peralta et al., 2000). For instance, to offer better resistance to desiccation during low tide, *Z. noltei* has a narrower leaf than *Z. marina*, as *Z. noltei* covers the large intertidal flats of Arcachon Bay while *Z. marina* only grows in submerged channels (Plus et al., 2010).

**TABLE 4:** This table shows the nodes that have undergone adjustment when transferring the existing DBN to the Arcachon Bay case study

Node	Definition	What has changed?
Accumulated Light	Probability of meeting light requirements for the normal function of the plant representing accumulated variations and effects in that month	The addition of a third state. The 2‐state and 3‐state models are compared
Genera Presence	Categorical, proportion of meadow of that genera.	The current model adds two specific *Zostera* species: *Z. marina* and *Z. noltei*
Physiological Status of Plants	The physiological status captures the degree to which the plant can function normally	Node modeled as a function of light factor—CPTs are adjusted when considering a 3‐state light model
Baseline Shoot Density	Best case expected shoot density for a given month given the physiological status of the meadow. Used to explicitly capture large seasonal variations	The CPTs are estimated for each species separately to capture the different growth strategies between species
Loss in Shoot Density	Loss in shoot density for that month	Node modeled as a function of light factor—CPTs are adjusted when considering a 3‐state light model
Seed Density	Density of seeds per *m* ^2^. States capture the dynamic range in growth rates from fast colonizing species to slow persistent species	The CPTs are adjusted to capture the reproduction cycle for the two species
Recruitment Rate from Seeds	Rate of recruitment into the adult population from seeds	The CPTs are adjusted to capture the reproduction cycle for the two species. Node modeled as a function of light factor CPTs adjusted when considering a 3‐state light model

*Note*: In addition, a definition of the nodes is provided and where the change took place in each node. Definition of the nodes is obtained from “Timing anthropogenic stressors to mitigate their impact on marine ecosystem resilience Supplementary Information” by Wu et al. (2017), nature communications 8:1263, Table S3.

#### 3.1.2 Knowledge acquisition

##### Step 2: Collect information

Our study had access to both seagrass data, but only limited data, and environmental experts with local knowledge. Therefore, since data was limited and insufficient to “learn” the DBN model structure, the effort to harness the expert knowledge to adapt the model became critical.

Overall, the main inputs for the model included the state probability for light (environmental), the genera and location‐specific parameters relating to climate (tropical or temperate), depth and tidal exposure (subtidal or intertidal), and transitory or enduring (persistent) type of meadow (site conditions). The key metric of interest to management was shoot density (number of shoots *m* ^2^). The light impact on seagrass ecosystems was considered in terms of ecological baselines and as a key stressor to modeling risk.

#### 3.1.3 Site application

##### Step 3: Review the CPTs

The nodes that required modification for the case study included those that characterized the seasonal growth and reproduction dynamics of the *Zostera* spp. The CPTs of the following nodes were updated: physiological status of plants, nodes used to capture seagrass growth dynamics (baseline shoot density, loss in shoot density), and seasonal variation in seagrass reproduction (seed density, and recruitment rate from seeds; Table 4). The seagrass growth captured via shoot density factor had the CPTs estimated separately for *Z. marina* and *Z. noltei* to capture the different growth strategies between the species. In contrast, the conditional probabilities of the elements used to represent the reproductive cycle of the seagrass, such as the seed density and seed recruitment rate factors, were also adjusted, but the CPTs computed for these parameters were the same for both species. That is because the seasonal variation in reproduction does not differ between the two species of *Zostera*. Furthermore, while assessing their CPTs for the 3‐state of light model, nodes utilized to reflect the influence of different light conditions on the seagrass population, including loss in shoot density, physiological status of plants, and seed recruitment rate, were modified (Table 4). This is because a third state was added to the light node.

##### Step 4: Scenario assessment

**FIGURE 5:**
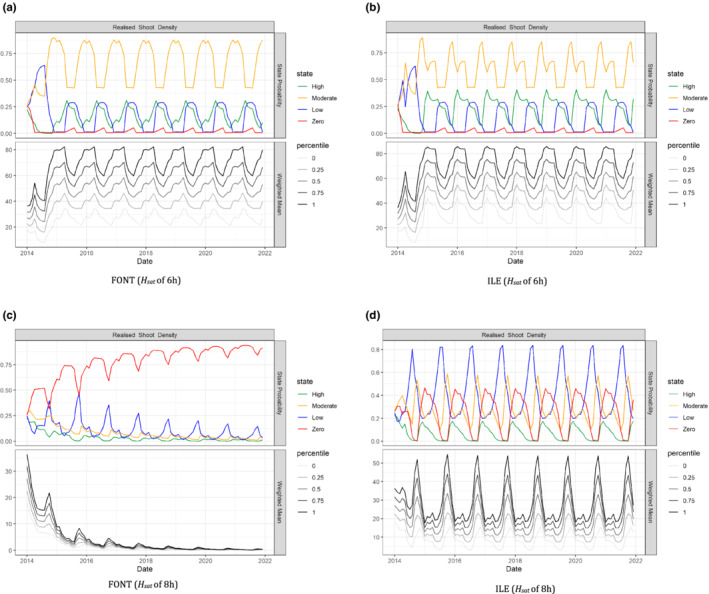
The model predicted‐state probabilities for shoot density for *Z. noltei* located at FONT and ILE. The initial 24 months are used for initialisation to allow the system to enter the baseline pattern. Top plots are the probability of each shoot density state, and the bottom plots show the weighted mean of the expected value and the interquartile range. Shoot density state probabilities for seagrass located at (a) FONT and (b) ILE, when considered *H*_sat_ of 6 h as light thresholds to estimate the light conditions used as input to the model. shoot density state probabilities for seagrass located at (c) FONT and (d) ILE, when considered *H*_sat_ of 8 h as light thresholds to estimate the light conditions used as input to the model.

In our case study, the model infers predicted‐state probabilities for shoot density based on scenarios of different species (*Z. marina* or *Z. noltei*), the light conditions (2‐ or 3‐state) and site‐specific parameters relating to depth and tidal exposure (subtidal or intertidal; Figure 5). In the absence of light thresholds data, we considered ranges of values based on expert judgments as evidence of light conditions. This process of varying the value of uncertainty one at a time while keeping all other factors fixed helped us to draw conclusions about whether it should have further adjustments.

Each subfigure comprises two panels, where the top panel shows the state probability trajectories over time for the states indicated, while the bottom panel shows the weighted mean response (assuming a uniform distribution) of the expected value and the interquartile range. As can be seen from Figure 5, a light saturation threshold *I*_k_ that is higher than available light leads to significant decline in shoot density but the level of impact differs by site. For example, when comparing FONT with ILE for *H*_sat_ = 8 h, the meadow is driven to zero shoot density for seagrasses located at FONT, while this pattern is not observed at ILE.

##### Step 5: Validate model performance

The model was validated by comparing simulated scenarios corresponding to unobserved parameters (i.e. light thresholds) with observed data (shoot density and light over time). When considering the *H*_sat_ of 6 h in the 2‐state model, the MSE in the predicted‐state probabilities compared to observed values was found to be of the order of 0.01 to 0.04 across the four sites (Table 5), demonstrating an acceptable fit of the model to the data. Furthermore, the ability of the model to predict seagrass shoot density trends was also validated for the 3‐state of light, in which the MSE values are on the order of 0.01 for GAIL and ILE for *H*_sat_ of 6 h and *H*_comp_ of 8.5 (Table 6). For the other two sites, FONT and ROCH, the lowest MSE estimated are 0.02 and 0.01, respectively, is observed when the highest light thresholds are considered. Thus, the 2‐ and 3‐state models demonstrated a similar ability to predict the trends for the *Z. noltei* at Arcachon Bay; nevertheless, because of parsimony and data limitations in a model transferability context, we decided to go with a 2‐states light model and *H*_sat_ of 6 h for Arcachon Bay.

**TABLE 5:** MSE for the 2‐state model per site (FONT, GAIL, ILE and ROCH) and considering different lengths of daily light periods thresholds (*H*_sat_, hours) for *Z. noltei*

*H*_sat_	FONT	GAIL	ILE	ROCH
4	0.0417	0.0401	0.0405	0.0424
5	0.0399	0.0392	0.0395	0.0409
5.5	0.0390	0.0387	0.0392	0.0403
6	0.0362	0.0145	0.0121	0.0183
7	0.0586	0.0176	0.0434	0.0490
7.5	0.1446	0.0923	0.0785	0.0712
8	0.1977	0.1158	0.0930	0.1327
8.5	0.2848	0.1965	0.1764	0.2553
9	0.2939	0.2904	0.2605	0.2855

*Note*: The warmer colours indicate higher values of MSE, and green colours indicate lower values of MSE.

**TABLE 6:** MSE for the 3‐state model per site (FONT, GAIL, ILE and ROCH) and considering different lengths of daily light periods thresholds (*H*_sat_ and *H*_comp_, hours) for *Z. noltei*

*H*_sat_	*H*_comp_	FONT	GAIL	ILE	ROCH
6	8.5	0.0254	0.0122	0.0108	0.0164
6	9	0.0295	0.0128	0.0113	0.0175
6	10	0.0336	0.0137	0.0116	0.0175
6	11	0.0363	0.0140	0.0117	0.0187
6	12	0.0363	0.0140	0.0117	0.0175
7	8.5	0.0230	0.0185	0.0168	0.0150
7	9	0.0269	0.0332	0.0287	0.0194
7	10	0.0416	0.0155	0.0423	0.0130
7	11	0.0543	0.0460	0.0425	0.0484
7	12	0.0561	0.0457	0.0427	0.0130
8	8.5	0.0822	0.0471	0.0348	0.0246
8	9	0.0875	0.0409	0.0365	0.0522
8	10	0.1495	0.0738	0.0775	0.0130
8	11	0.1976	0.1032	0.0846	0.1013
8	12	0.2021	0.1239	0.0910	0.0130
8.5	8.5	0.1258	0.0442	0.0418	0.0380
8.5	9	0.1241	0.0504	0.0450	0.0845
8.5	10	0.1887	0.1071	0.0938	0.0130
8.5	11	0.2669	0.1748	0.1263	0.2028
8.5	12	0.2758	0.1869	0.1782	0.0130
9	8.5	0.1247	0.0507	0.0455	0.0604
9	9	0.1356	0.0835	0.1020	0.0990
9	10	0.2045	0.1279	0.1160	0.0130
9	11	0.2798	0.2204	0.1717	0.2348
9	12	0.2911	0.2421	0.2072	0.0130

*Note*: The warmer colours indicate higher values of MSE, and green colours indicate lower values of MSE.

## 4 DISCUSSION

Model transferability and adaptation can be highly beneficial, since methods to enable reusing and adapting models can help with widespread model uptake to support managers and decision makers, especially for sites with limited data. In general, transferring a model to a new context can shorten the time and effort to develop a new model by adapting an existing model. Although not a replacement for comprehensive data and studies, model transferability helps to provide predictive evidence on potential future scenarios to support proactive management, such as in the management of resilience. This paper has demonstrated the transferability of an existing general seagrass ecosystem DBN model to new sites and offered guidelines on model transferability that could be applicable across different contexts and scales around the world.

In the future, substantial losses of seagrass meadows are expected in response to human impact, both through direct proximal impacts affecting seagrass meadows locally and indirect impacts, which may affect seagrass meadows far away from the sources of the disturbance (Duarte, 2002). Thus, the ability to transfer a global model and concepts and apply them to a local case study can help protect and sustainably manage these valuable marine resources such as the seagrass meadows located in Arcachon Bay.

One of the challenges we faced in the study arose in defining the light thresholds to characterize the regional light regime and the lack of extensive empirical data available to validate our model. Although we have shown that applying such a range of different light thresholds provides valuable insights into the effects of light intensity and duration variability on seagrass ecosystems, determining an appropriate light threshold for seagrasses involves several challenges. For example, light requirements are unknown for many seagrass species, particularly locally‐specific thresholds. The light levels can differ over multiple timescales; seagrass light requirements may vary by season and a range of environmental parameters, including water temperature and sediment chemistry (Koch, 2001; Lee et al., 2007). Furthermore, the levels of adaptability of the plants to respond to changing environmental conditions can differ among species (Collier et al., 2012).

Bayesian inference necessitates the use of certain prior distributions. Hence, approaches concerned with choosing a proper prior for a statistical analysis have been developed (Kass & Wasserman, 1996; Sarma & Kay, 2020). Expert informed priors have been used in BN models to help ecologists go from conceptual models to statistical models that are calibrated to observed data (O'Leary et al., 2009). Generally, experienced experts translate what is known about an application into choosing a probability distribution by reflecting beliefs about the unknown values of certain quantities. For example, expert knowledge was utilized to fill in data gaps for a model of distribution of the brush‐tailed rock‐wallaby (*Petrogale penicillatus*) (Murray et al., 2009). Prior probability distributions expressing what is known about a particular model parameter are easily included in Bayesian approaches (Gelman, 2003) because they may be obtained from earlier studies or built on expert knowledge (Garthwaite & O'Hagan, 2000; Gelman, 2003; Kuhnert et al., 2010). Substantial research on the conservation science and ecological literature details the application of these methods (Kuhnert et al., 2005; Martin et al., 2005).

Wang et al. (2018) developed effective numerical methods in which history matching specifies a prior distribution from expert‐elicited information. As a result, a set of appropriate prior choices can be used as a basis for making a unique prior choice less arbitrary in a sensitivity analysis (Wang et al., 2018). Based on that, an alternative model updating approach is also outlined here (see Supporting Information S2) to apply the calibration of light thresholds, and identify which best light model and threshold fit the empirical data. Although discretization thresholds can be drawn from experts and literature when there is limited or no data available, finding high‐scoring discretization is difficult or impractical due to a large number of possibilities that need to be verified, which makes this approach beneficial. This methodology has the potential to be particularly valuable to select optimum DBN inputs (e.g., light thresholds) in data‐scarce regions.

Another challenge faced in this project was the scarce data to validate the model and the balance between a more detailed model and a practical model that is supported by available data and expert knowledge. For example, discretising the light parameter into three states instead of two did not show better estimates for shoot density values when compared to the data. Furthermore, as there was only data for one species, steps 1–5 were only achievable for *Z. noltei*, whereas for *Z. marina*, it was only possible to complete steps 1–3 due to data limitations (Figure 1). Such a systematic set of guidelines can additionally help modelers and experts to identify potential limitations in the scope of the developed models, and where more study and data is needed. Although we focused on transfer of a general DBN to a local site and species, it could also include transfers to other stressors. For example, stressors from new environmental hazards or climate stress, such as heat stress caused by marine heatwaves, can be included in the model to explore changes in seagrass response.

## 5 CONCLUSIONS

Model users are increasingly transferring models to alternative sites where data can be scarce. When transferring a model from one context to a new application context, the effort in developing a model is reduced, and data collection can be less demanding. In this regard, models transferred to novel conditions could provide predictions in data‐poor scenarios, contributing to more informed management decisions. In this study, we have demonstrated the transferability of an existing general seagrass ecosystem DBN model to new sites and offered general guidelines capturing the lessons learned here. Moreover, the DBN adapted for the Arcachon Bay case study can also be applied to various other domains in ecology. For example, other stressors can be incorporated into the model, such as effects caused by climate events, to explore changes in seagrass response.

## AUTHOR CONTRIBUTIONS

**Paula Sobenko Hatum:** Conceptualization (equal); data curation (lead); formal analysis (equal); investigation (equal); methodology (equal); resources (equal); validation (equal); visualization (equal); writing – original draft (lead); writing – review and editing (lead). **Paul Pao‐Yen Wu:** Conceptualization (equal); data curation (equal); formal analysis (supporting); investigation (equal); methodology (equal); resources (equal); supervision (lead); validation (equal); visualization (equal); writing – original draft (supporting); writing – review and editing (supporting). **Kathryn McMahon:** Conceptualization (equal); data curation (equal); formal analysis (supporting); investigation (equal); methodology (supporting); resources (equal); supervision (supporting); validation (equal); visualization (equal); writing – original draft (supporting); writing – review and editing (supporting). **Kerrie Mengersen:** Conceptualization (equal); data curation (supporting); formal analysis (supporting); investigation (supporting); methodology (supporting); resources (equal); supervision (supporting); validation (supporting); visualization (equal); writing – original draft (supporting); writing – review and editing (supporting).

## CONFLICT OF INTEREST

The authors declare that they have no competing interests.

## Supporting information


**Appendix S1.** K‐Nearest Algorithm Used to Determine Ik and Ic
**Appendix S2.** Prior predictive approach ‐ An alternative approach to calibrating the model
**Appendix S3.** Supporting Figures
**Appendix S4.** Supporting Tables

## Data Availability

Validation data used to validate the DBN model for Zostera noltei at Arcachon Bay, France is provided in supporting information, supporting tables, Table S6 (doi: 10.5061/dryad.7m0cfxpxd).
