# Supplementary material for: Guidelines for model adaptation: A study of the transferability of a general seagrass ecosystem Dynamic Bayesian Networks model
Source: Ecol Evol. 2022 Aug 4;12(8):e9172. doi: 10.1002/ece3.9172 (PMC9353019; doi:10.1002/ece3.9172)
Supplement: Supplementary file 1 — Appendix S1. K‐Nearest Algorithm Used to Determine Ik and Ic Appendix S2. Prior predictive approach ‐ An alternative approach to calibrating the model Appendix S3. Supporting Figures Appendix S4. Supporting Tables [file ECE3-12-e9172-s001.docx]

# Supporting Information

## S1. K-Nearest Algorithm Used to Determine *I_k_* and *I_c_*

Here we show the nearest neighbour algorithm utilised to obtain the seasonal *I_k_* and *I_k_* values for Arcachon Bay. The data we have available is the average water temperature in February, May, July and December of 4-day samplings in Thao Lagoon, Mediterranean, a nearby area of Arcachon bay. Besides the temperature, the respective *I_k_* and *I_c_* values for those months are shown in Table 1 for *Z. marina* and *Z. noltei*. The average monthly water temperature and the *I_k_* and *I_c_* estimated for *Z. marina* and *Z. noltei* for the nine sites of the Bay are summarised in Table S2 and S3.

From the KNN approach, we used the average temperature obtained for Arcachon Bay as a form of proximity survey to find its closest neighbour to the Thao Lagoon data in terms of the temperature (Fix and Hodges, 1989). For example, suppose *T*_1_ is the temperature value in Arcachon Bay, for which *I_k_* and *I_c_* need to be estimated. First, we find the one closest point to *T*_1_ and then the *I_k_* and *I_c_* values associated with the nearest point are assigned to *T*_1_.

## S2. Prior predictive approach - An alternative approach to calibrating the model

Both 2 and 3-state models include above saturation and below saturation state; however, the 3-state model has the addition of a third light state, named below limitation. To incorporate light data in DBN inference via states of above, below and/or below limitation light, the probability of being in one of these states is based on light intensity and light duration per day. As light thresholds were not well understood in our study area, a prior predictive simulation approach (Wang et al., 2018) can be implemented to identify the thresholds which best fit the empirical data. Under this approach, prior distributions are placed on each parameter that determines the number of states and the thresholds. Then, a set of parameters is drawn from these priors and used to create a hypothetical dataset from the model. If this prior predictive dataset is sufficiently close to the observed data for pre-determined metrics, the prior values are considered plausible (Hartmann et al., 2020). This ABC process is repeated many times, resulting in a distribution of a reasonable number of states and thresholds for light.

The general workflow for the prior predictive simulation approach can be described as composing these steps:

1. Draw the number of states *K* from prior distributions.
2. Given *K*, draw a set of values of light thresholds T to the respect parameters (*H_sat_* and *Hcomp*).
3. Given the values of *K* and *T* simulate the dataset from the model.
4. Use Approximate Bayesian Computation (ABC) approach to compare simulated datasets and observed data.

The first step is to choose a probability distribution for each prior and then draw *N* values from different prior distributions. The number of states can be assumed as *k* ∼ *Categorical*(2*,*3), with each category having a probability of 0.5. If *K* = 2, *T* = *T_sat_* and assumed as *T_sat_* ∼ *Uniform*(4*,*9), while if *K* = 3 − *sates*, *T* = *T_sat_,T_comp_* where *T_sat_* is assume as *T_sat_* ∼ *Uniform*(4*,*9) and *T_comp_* as *T_comp_* ∼ *Uniform*(8*.*5*,*12).

The goal was to use these reference points to find a joint prior distribution that produced reasonable values of potential seagrass shoot density. Formally, we want to know the density *p*(·) of data points *y_pred_,...,y_predN_* from a dataset *y_pred_* of length *N*, given a vector of priors and the likelihood (·*/*Θ) (in this study, Θ = *K,T*) before seeing any observations. Here, *y_pred_* represents the predicted-states probabilities for shoot density. After simulating from these priors, the ABC method is used to check if the *y_pred_* produces the simulations that match the observed dataset. If the simulated dataset differs substantially from the observed data, this sample should not be considered as the estimate of the parameter. Conversely, if a sample of the unknown parameter produces the simulation that matches the observed dataset, this sample should be close to the exact value of the parameter.

# Supporting Figures

## Figure S1

Example of a framework used to review the model structure using expert knowledge. The tasks of the research team and experts are shown in A) and B), respectively.


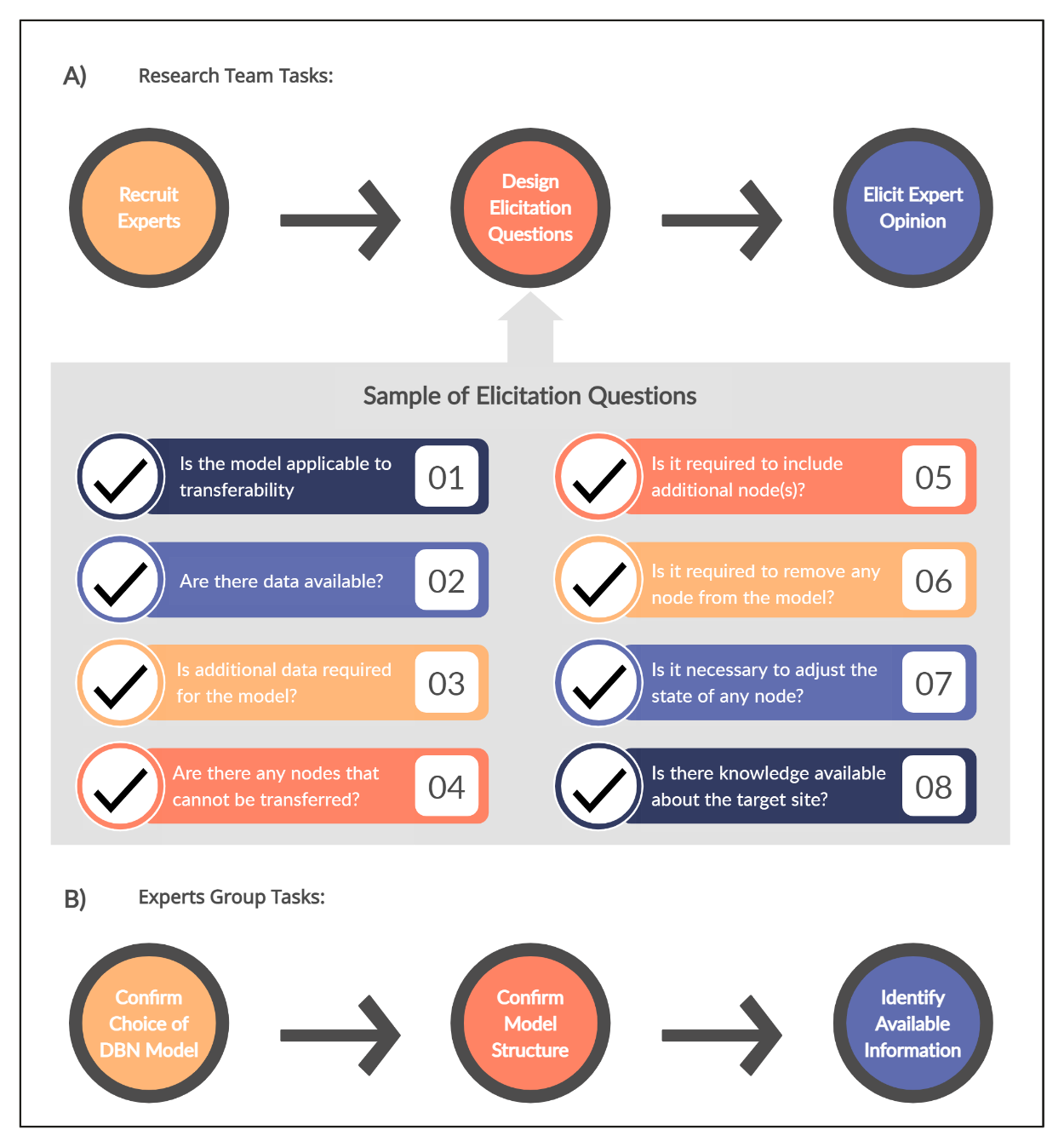


## Figure S2a

The overall Dynamic Bayesian Network (DBN) network structure. Nodes are ovals and arrows denote causal parent-child relationships in the direction of the arc where a parent node (e.g., Meadow Type) influences a child node (e.g., Location Type); conversely, an absence of a link implies conditional independence. Rounded rectangles denote subnetworks, i.e., networks that describe a component of the system, expressed as a single node in the larger BN (object-oriented approach) (Johnson and Mengersen, 2012). Nodes are coloured as follows: white for site condition nodes, purple for recovery nodes, green for resistance nodes, blue for environmental nodes, yellow for population (shoot density) nodes, and pink for all other nodes. Figure adapted from “Timing anthropogenic stressors to mitigate their impact on marine ecosystem resilience Supplementary Information” by Wu et al. (2017), Nature Communications 8:1263, Supplementary material, Figure 7. The figure was developed using GeNIe, a development platform for creating graphical decision theoretic models. The following symbology is used in this figure:

1. **A node with a check mark:** A node is ticked when inference has been successfully executed.
2. **A curved arrow back onto itself:** Denotes a link to the node’s next time slice (i.e., *t*+1).
3. **A double-headed arrow:** Indicates that there are arcs heading both ways between two subnetworks. For example, a double-headed arrow between a node N and a subnetwork S means that there is at least one node in S that depends on N and that there is at least one node in S that influences N.
4. **An arrow labelled with a [1]:** Indicates a connection to a subsequent time slice (*t* + 1).
5. **An arrow labelled with an orange [1] and a green [1]:** There is no difference between arrows labelled with orange [1] and green [1]. They all denote a connection to a subsequent time slice (*t* + 1).
6. **Arrow colour (i.e., grey arrows vs. blue arrows vs. light blue–blue arrows):** In this figure, the varying arrow colours have no particular meaning.


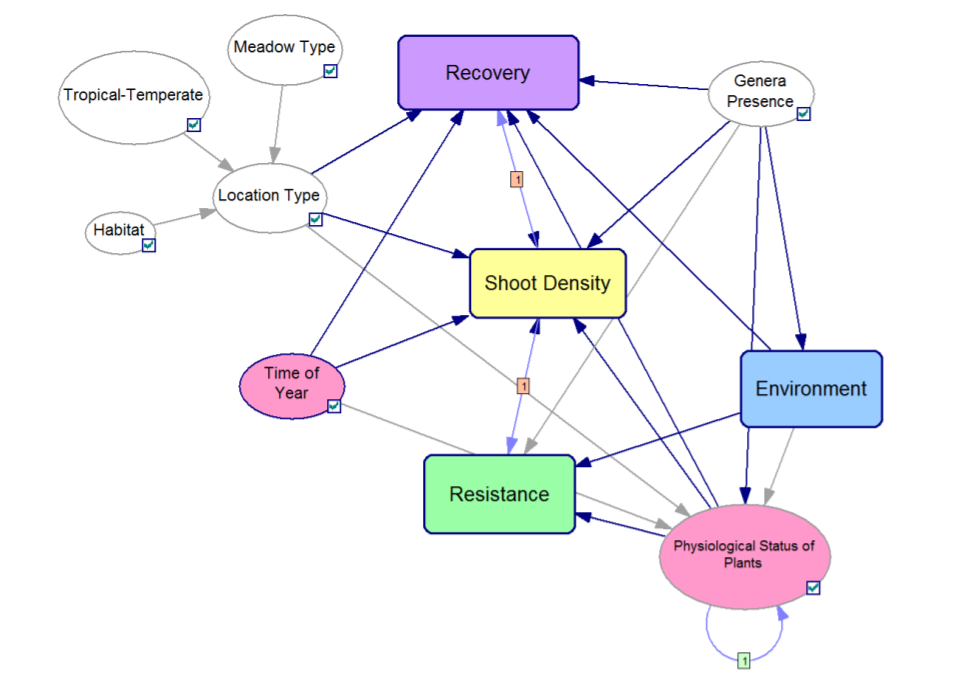


## Figure S2b

Illustration of time slicing in the DBN for Physiological status of plants of genus *Zostera*, which are persistent, live in a temperate climate, and intertidal zone. It is considered that the plants initially present a (I) good and (II) poor physiological state.


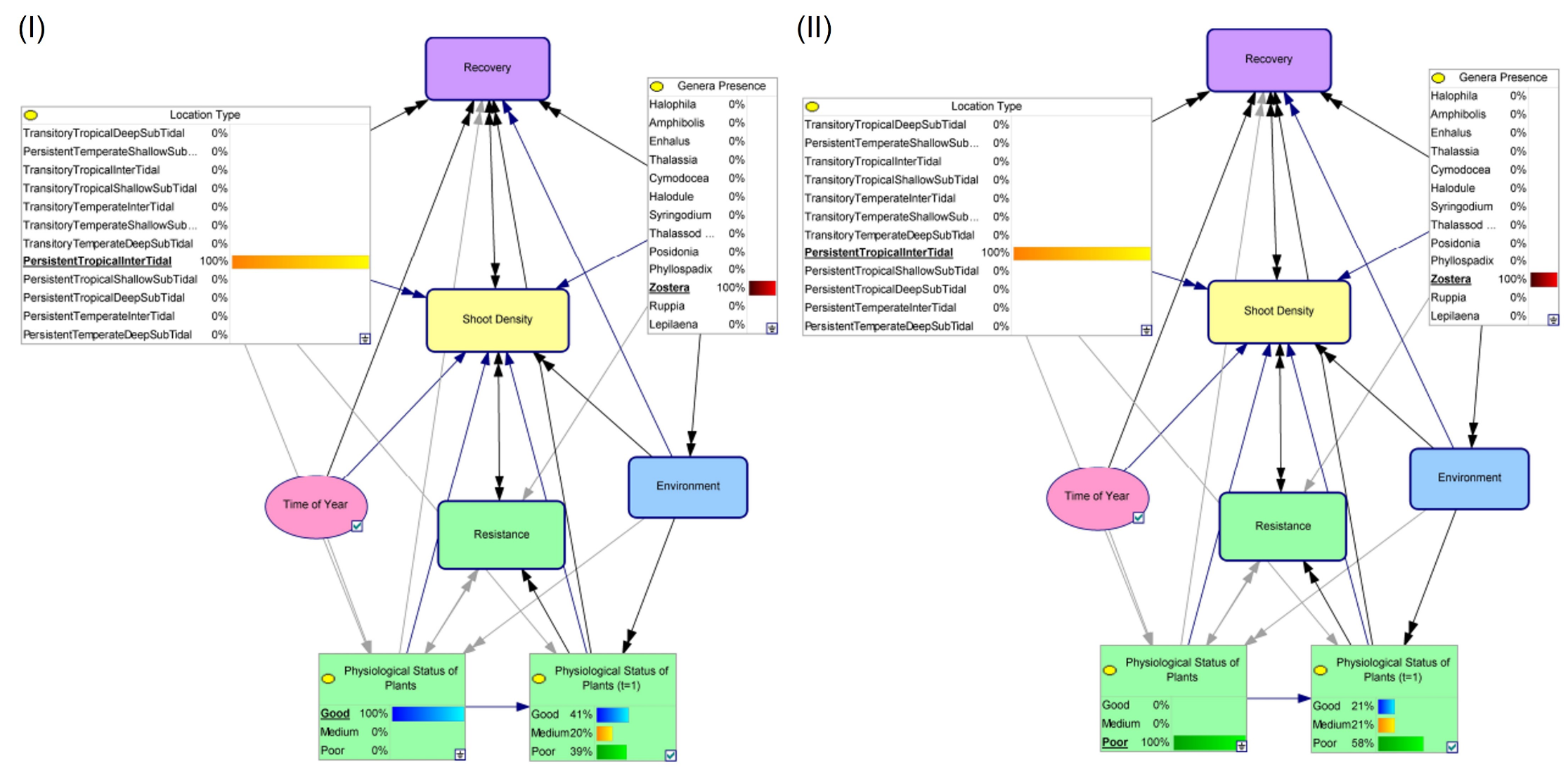


# Supporting Tables

## Table S1

Expert involvement in elicitation and validation along with their area of expertise.

| **Experts** | **Category of**  **Experts** | **Expertise** | **Step 1** | **Step 2** | **Step 3** | **Step 4** |
| --- | --- | --- | --- | --- | --- | --- |
| Etienne Auclair | General | Marine Ecology | Yes | Yes | Yes | Yes |
| Florian Ganthy | General | Marine Ecology | Yes | Yes | Yes | Yes |
| Heloise Muller | General | Marine Ecology | Yes | Yes | Yes | Yes |
| Isabelle Auby | Specialist | Seagrass | Yes | Yes | Yes | Yes |
| Julian Caley | General | Marine Ecology | Yes | Yes | Yes | Yes |
| Kathryn McMahon | Specialist | Seagrass | Yes | Yes | Yes | Yes |
| Martin Marzhon | General | Marine Ecology | Yes | Yes | Yes | Yes |
| Martin Plus | Specialist | Seagrass | Yes | Yes | No | No |

## Table S2

List of studies comparing modelling approaches. The table indicates the context and objective of the study, the algorithm/approach used, and the main findings from this research.

| **Study** | **Context** | **Objective** | **Approach** | **Main findings** |
| --- | --- | --- | --- | --- |
| Wenger and Olden (2012) | Ecological models | Propose a method to  evaluate transferability | GLMM, ANN,  RF classifier | Simpler models can be more transferable |
| Moon et al. (2017) | Ecological models | Propose a method of describing a model’s  application niche for  use during the model selection process | Model performance curves and heat maps | Synthesize information from databases, past studies, and/or  past model transfers to create  model performance curves and heat maps |
| Sequeira et al. (2018) | Ecological models | Provide guidelines for transferring  biodiversity models | Synthesize current advances in biodiversity model transfer | Propose a set of guidelines to support efficient learning and the improvement of model transferability |
| Vanreusel, Maes and Van Dyck (2007) | Species distribution models (SDMs) | Test the transferability of habitat-based predictive distribution models | GLM | Habitat models based on essential functional resources  could transfer better in space  than models that use indirect environmental variables. |
| Barbosa, Real and Vargas (2009) | Species distribution models (SDMs) | Test hypotheses: (1) Data completeness is more  important than the predictor  range; (2) predictor range  is more important than data completeness | The favourability function of  Real, Barbosa and Vargas (2006),  GLM | The range of values of the predictors is more important than data completeness |
| Rapacciuolo et al. (2012) | Species distribution models (SDMs) | Test if climate-based correlative SDMs can  predict distribution alterations | ANN, CTA, GAM, GBM,  GLM, MARS, MAXENT,  RF, SRE | Maxent performed better than  GLM and RF |
| Heikkinen, Marmion and Luoto (2012) | Species distribution models (SDMs) | Compare the performance of different model types  in interpolative validation  and extrapolative validation,  and compare among the three species groups | GAM, GLM, MARS,  MAXENT, GBM, ANN,  RF, GARP, CTA, MDA | Maxent, GLMs, and GLMs considered the most transferable methods |
| Moreno-Amat et al. (2015) | Species distribution models (SDMs) | Assess the effect of model complexity on the performance of Maxent | MAXENT | The number of variables used can impact on model transferability |
| Morán‐Ordóñez et al. (2017) | Species distribution models (SDMs) | Provide an assessment of the predictive performance of SDMs over time | MAXENT | Data quality has a considerably higher influence on model  performance than that of species traits |
| Bridge et al. (2020) | Species distribution models (SDMs) | Predict the presence or absence of a taxon outside the modelled region | BRTs | Models developed in one location accurately predicted the presence and absence of the same taxa in another region. |
| Randin et al. (2006) | Ecological niche models (ENM) | Assess the geographical transferability of ENMs  fitted with two modelling  techniques | GAM, GLM | GAM performed better than GLM |
| Peterson, Papes and Eaton (2007) | Ecological niche models (ENM) | Compare predictive success in two common algorithms | GARP, MAXENT | Maxent performed better than GLM and RF |
| Sequeira et al. (2016) | Ecological niche models (EN) | How to improve  transferability of predictive models of fish species richness on coral reefs | GLM, GLMM | When calibration data and evaluation data have similar  spatio-temporal scales, models presented better transferability |
| Strauss and Biedermann (2007) | Habitat models | Transfer models to test data from another year data | GLMs | Models built on the larger dataset are more general than |
| Tuanmu et al. (2011) | Habitat models | from different locations  Assess how different satellite-derived  variables affect temporal transferability | MAXENT | the ones basing on the smallerMulti-year phenology metrics  derived from MODISdataset  reduce model complexity and multicollinearity among  predictor variables improving model transferability |
| Lauria et al. (2015) | Habitat models | Provide a modelling framework to  investigate the habitat preference of nine elasmobranch species | GAM | A framework that includes four steps: 1) model selection,  2) model evaluation, 3) model transferability between areas and 4) model mapping |

## Table S3

Average water temperature (Temp, °C), saturation and compensation irradiance (*I_k_* and *I_c_*, µmols m^−2^ s^−1^) estimated for *Z. marina* and *Z. noltei* located at Thao Lagoon. From “Seasonal variations in photosynthetic irradiance response curves of macrophytes from a Mediterranean coastal lagoon” by Plus et al., 2005, Aquatic Botany 81 (2): 157-173.

|  |  | **Feb** | **May** | **Jul** | **Dec** |
| --- | --- | --- | --- | --- | --- |
| ***Z. marina*** | **Temp** | 7 | 17 | 25 | 13 |
|  | ***I_k_*** | 70 | 245 | 501 | 99 |
|  | ***I_c_*** | 19 | 53 | 395 | 40 |
| ***Z. noltei*** | **Temp** | 7 | 17 | 25 | 13 |
|  | ***I_k_*** | 222 | 305 | 254 | 174 |
|  | ***I_c_*** | 72 | 35 | 33 | 19 |

## Table S4

Average water temperature (Temp, °C), saturation and compensation irradiance (*I_k_* and *I_c_*, µmols m^−2^ s^−1^) estimated for *Z. marina* located at ANDE, FONT, GAIL, GARR, HAUT, ILE, JACQ, PASS and ROCH.

|  |  | **Jan** | **Feb** | **Mar** | **Apr** | **May** | **Jun** | **Jul** | **Aug** | **Sep** | **Oct** | **Nov** | **Dec** |
| --- | --- | --- | --- | --- | --- | --- | --- | --- | --- | --- | --- | --- | --- |
| **ANDE** | **Temp** | 11 | 11 | 14 | 16 | 19 | 22 | 25 | 26 | 24 | 18 | 14 | 13 |
|  | ***I_k_*** | 99 | 99 | 99 | 245 | 245 | 501 | 501 | 501 | 501 | 245 | 99 | 99 |
|  | ***I_c_*** | 40 | 40 | 40 | 53 | 53 | 395 | 395 | 395 | 395 | 53 | 40 | 40 |
| **FONT** | **Temp** | 11 | 11 | 13 | 16 | 20 | 23 | 26 | 27 | 24 | 17 | 14 | 12 |
|  | ***I_k_*** | 99 | 99 | 99 | 245 | 245 | 501 | 501 | 501 | 501 | 245 | 99 | 99 |
|  | ***I_c_*** | 40 | 40 | 40 | 53 | 53 | 395 | 395 | 395 | 395 | 53 | 40 | 40 |
| **GAIL** | **Temp** | 11 | 11 | 13 | 16 | 19 | 22 | 25 | 25 | 23 | 18 | 14 | 12 |
|  | ***I_k_*** | 70 | 70 | 99 | 99 | 245 | 245 | 501 | 501 | 501 | 245 | 99 | 99 |
|  | ***I_c_*** | 20 | 20 | 40 | 40 | 53 | 53 | 395 | 395 | 395 | 53 | 40 | 40 |
| **GARR** | **Temp** | 11 | 11 | 12 | 15 | 18 | 22 | 24 | 24 | 22 | 17 | 14 | 11 |
|  | ***I_k_*** | 99 | 99 | 99 | 245 | 245 | 501 | 501 | 501 | 501 | 245 | 99 | 99 |
|  | ***I_c_*** | 40 | 40 | 40 | 53 | 53 | 395 | 395 | 395 | 395 | 53 | 40 | 40 |
| **HAUT** | **Temp** | 11 | 11 | 13 | 16 | 19 | 23 | 25 | 25 | 23 | 17 | 14 | 12 |
|  | ***I_k_*** | 99 | 99 | 99 | 245 | 245 | 501 | 501 | 501 | 501 | 245 | 99 | 99 |
|  | ***I_c_*** | 40 | 40 | 40 | 53 | 53 | 395 | 395 | 395 | 395 | 53 | 40 | 40 |
| **ILE** | **Temp** | 12 | 12 | 14 | 16 | 19 | 23 | 25 | 25 | 24 | 19 | 15 | 13 |
|  | ***I_k_*** | 99 | 99 | 99 | 245 | 245 | 501 | 501 | 501 | 501 | 245 | 245 | 99 |
|  | ***I_c_*** | 40 | 40 | 40 | 53 | 53 | 395 | 395 | 395 | 395 | 53 | 53 | 40 |
| **JACQ** | **Temp** | 11 | 11 | 13 | 15 | 19 | 22 | 24 | 25 | 23 | 17 | 14 | 12 |
|  | ***I_k_*** | 99 | 99 | 99 | 245 | 245 | 501 | 501 | 501 | 501 | 245 | 99 | 99 |
|  | ***I_c_*** | 40 | 40 | 40 | 53 | 53 | 395 | 395 | 395 | 395 | 53 | 40 | 40 |
| **PASS** | **Temp** | 11 | 11 | 13 | 16 | 19 | 22 | 25 | 26 | 23 | 18 | 14 | 12 |
|  | ***I_k_*** | 99 | 99 | 99 | 245 | 245 | 501 | 501 | 501 | 501 | 245 | 99 | 99 |
|  | ***I_c_*** | 40 | 40 | 40 | 53 | 53 | 395 | 395 | 395 | 395 | 53 | 40 | 40 |
| **ROCH** | **Temp** | 12 | 11 | 13 | 16 | 19 | 23 | 25 | 26 | 24 | 18 | 14 | 12 |
|  | ***I_k_*** | 99 | 99 | 99 | 245 | 245 | 501 | 501 | 501 | 501 | 245 | 99 | 99 |
|  | ***I_c_*** | 40 | 40 | 40 | 53 | 53 | 395 | 395 | 395 | 395 | 53 | 40 | 40 |

## Table S5

Average water temperature (Temp, °C), saturation and compensation irradiance (*I_k_* and *I_c_*, µmols m^−2^ s^−1^) estimated for *Z. noltei* located at ANDE, FONT, GAIL, GARR, HAUT, ILE, JACQ, PASS and ROCH.

|  |  | **Jan** | **Feb** | **Mar** | **Apr** | **May** | **Jun** | **Jul** | **Aug** | **Sep** | **Oct** | **Nov** | **Dec** |
| --- | --- | --- | --- | --- | --- | --- | --- | --- | --- | --- | --- | --- | --- |
| **ANDE** | **Temp** | 11 | 11 | 14 | 16 | 19 | 22 | 25 | 26 | 24 | 18 | 14 | 13 |
|  | ***I_k_*** | 174 | 174 | 174 | 305 | 305 | 254 | 254 | 254 | 254 | 305 | 174 | 174 |
|  | ***I_c_*** | 19 | 19 | 19 | 35 | 35 | 33 | 33 | 33 | 33 | 35 | 19 | 19 |
| **FONT** | **Temp** | 11 | 11 | 13 | 16 | 20 | 23 | 26 | 27 | 24 | 17 | 14 | 12 |
|  | ***I_k_*** | 174 | 174 | 174 | 305 | 305 | 254 | 254 | 254 | 254 | 305 | 174 | 174 |
|  | ***I_c_*** | 19 | 19 | 19 | 35 | 35 | 33 | 33 | 33 | 33 | 35 | 19 | 19 |
| **GAIL** | **Temp** | 11 | 11 | 13 | 16 | 19 | 22 | 25 | 25 | 23 | 18 | 14 | 12 |
|  | ***I_k_*** | 174 | 174 | 174 | 305 | 305 | 254 | 254 | 254 | 254 | 305 | 174 | 174 |
|  | ***I_c_*** | 19 | 19 | 19 | 35 | 35 | 33 | 33 | 33 | 33 | 35 | 19 | 19 |
| **GARR** | **Temp** | 11 | 11 | 12 | 15 | 18 | 22 | 24 | 24 | 22 | 17 | 14 | 11 |
|  | ***I_k_*** | 174 | 174 | 174 | 305 | 305 | 254 | 254 | 254 | 254 | 305 | 174 | 174 |
|  | ***I_c_*** | 19 | 19 | 19 | 35 | 35 | 33 | 33 | 33 | 33 | 35 | 19 | 19 |
| **HAUT** | **Temp** | 11 | 11 | 13 | 16 | 19 | 23 | 25 | 25 | 23 | 17 | 14 | 12 |
|  | ***I_k_*** | 174 | 174 | 174 | 305 | 305 | 254 | 254 | 254 | 254 | 305 | 174 | 174 |
|  | ***I_c_*** | 19 | 19 | 19 | 35 | 35 | 33 | 33 | 33 | 33 | 35 | 19 | 19 |
| **ILE** | **Temp** | 12 | 12 | 14 | 16 | 19 | 23 | 25 | 25 | 24 | 19 | 15 | 13 |
|  | ***I_k_*** | 174 | 174 | 174 | 305 | 305 | 254 | 254 | 254 | 254 | 305 | 305 | 174 |
|  | ***I_c_*** | 19 | 19 | 19 | 35 | 35 | 33 | 33 | 33 | 33 | 35 | 35 | 19 |
| **JACQ** | **Temp** | 11 | 11 | 13 | 15 | 19 | 22 | 24 | 25 | 23 | 17 | 14 | 12 |
|  | ***I_k_*** | 174 | 174 | 174 | 305 | 305 | 254 | 254 | 254 | 254 | 305 | 174 | 174 |
|  | ***I_c_*** | 19 | 19 | 19 | 35 | 35 | 33 | 33 | 33 | 33 | 35 | 19 | 19 |
| **PASS** | **Temp** | 11 | 11 | 13 | 16 | 19 | 22 | 25 | 26 | 23 | 18 | 14 | 12 |
|  | ***I_k_*** | 174 | 174 | 174 | 305 | 305 | 254 | 254 | 254 | 254 | 305 | 174 | 174 |
|  | ***I_c_*** | 19 | 19 | 19 | 35 | 35 | 33 | 33 | 33 | 33 | 35 | 19 | 19 |
| **ROCH** | **Temp** | 12 | 11 | 13 | 16 | 19 | 23 | 25 | 26 | 24 | 18 | 14 | 12 |
|  | ***I_k_*** | 174 | 174 | 174 | 305 | 305 | 254 | 254 | 254 | 254 | 305 | 174 | 174 |
|  | ***I_c_*** | 19 | 19 | 19 | 35 | 35 | 33 | 33 | 33 | 33 | 35 | 19 | 19 |

## Table S6

Data derived state probabilities for *Z. noltei* monitoring study (Cognat et al., 2018). Shoot density was observed at four sites in this study.

| **Site** | **Month** | **Shoot Density** | **Zero** | **Low** | **Moderate** | **High** |
| --- | --- | --- | --- | --- | --- | --- |
| **ILE** | January | 2868.61 | 0.12 | 0.15 | 0.63 | 0.10 |
| **GAIL** | January | 5125.88 | 0.13 | 0.16 | 0.62 | 0.10 |
| **ROCH** | January | 7900.43 | 0.13 | 0.15 | 0.62 | 0.10 |
| **FONT** | January | 6724.77 | 0.13 | 0.16 | 0.61 | 0.10 |
| **ILE** | February | 5596.14 | 0.20 | 0.20 | 0.53 | 0.07 |
| **GAIL** | February | 4655.61 | 0.21 | 0.20 | 0.53 | 0.06 |
| **ROCH** | February | 10392.83 | 0.21 | 0.21 | 0.53 | 0.06 |
| **FONT** | February | 6442.62 | 0.22 | 0.21 | 0.51 | 0.06 |
| **ILE** | March | 4890.74 | 0.24 | 0.22 | 0.48 | 0.05 |
| **GAIL** | March | 3621.03 | 0.24 | 0.22 | 0.49 | 0.05 |
| **ROCH** | March | 8652.86 | 0.24 | 0.22 | 0.49 | 0.05 |
| **FONT** | March | 9029.07 | 0.26 | 0.21 | 0.48 | 0.05 |
| **ILE** | April | 4561.56 | 0.21 | 0.20 | 0.53 | 0.06 |
| **GAIL** | April | 6771.80 | 0.20 | 0.20 | 0.54 | 0.07 |
| **ROCH** | April | 11098.23 | 0.21 | 0.21 | 0.52 | 0.06 |
| **FONT** | April | 12885.23 | 0.22 | 0.21 | 0.51 | 0.06 |
| **ILE** | May | 8135.57 | 0.13 | 0.15 | 0.63 | 0.09 |
| **GAIL** | May | 12132.81 | 0.12 | 0.15 | 0.63 | 0.10 |
| **ROCH** | May | 18528.40 | 0.13 | 0.15 | 0.62 | 0.10 |
| **FONT** | May | 22995.91 | 0.14 | 0.14 | 0.61 | 0.10 |
| **ILE** | July | 14107.92 | 0.03 | 0.04 | 0.59 | 0.34 |
| **GAIL** | July | 18575.42 | 0.03 | 0.05 | 0.59 | 0.33 |
| **ROCH** | July | 22666.72 | 0.03 | 0.05 | 0.59 | 0.32 |
| **FONT** | July | 19610.01 | 0.03 | 0.05 | 0.60 | 0.32 |
| **ILE** | August | 16553.29 | 0.02 | 0.03 | 0.49 | 0.47 |
| **GAIL** | August | 18152.19 | 0.02 | 0.03 | 0.48 | 0.47 |
| **ROCH** | August | 15988.97 | 0.02 | 0.03 | 0.49 | 0.46 |
| **FONT** | August | 12179.84 | 0.02 | 0.02 | 0.49 | 0.46 |
| **ILE** | August | 14813.31 | 0.02 | 0.03 | 0.49 | 0.46 |
| **GAIL** | August | 17587.87 | 0.02 | 0.03 | 0.48 | 0.47 |
| **ROCH** | September | 13919.81 | 0.02 | 0.03 | 0.44 | 0.52 |
| **FONT** | September | 15377.63 | 0.02 | 0.03 | 0.44 | 0.52 |
| **ILE** | September | 12650.10 | 0.02 | 0.02 | 0.44 | 0.53 |
| **GAIL** | September | 18716.50 | 0.01 | 0.02 | 0.43 | 0.53 |
| **ROCH** | September | 11991.73 | 0.01 | 0.02 | 0.44 | 0.52 |
| **FONT** | September | 14201.97 | 0.02 | 0.03 | 0.45 | 0.51 |
| **ILE** | October | 14201.97 | 0.02 | 0.03 | 0.49 | 0.46 |
| **GAIL** | October | 16600.32 | 0.02 | 0.03 | 0.48 | 0.47 |
| **ROCH** | October | 8840.96 | 0.02 | 0.03 | 0.50 | 0.46 |
| **FONT** | October | 12367.94 | 0.02 | 0.03 | 0.50 | 0.46 |
| **ILE** | November | 10251.75 | 0.03 | 0.04 | 0.59 | 0.34 |
| **GAIL** | November | 15189.52 | 0.03 | 0.05 | 0.59 | 0.33 |
| **ROCH** | November | 12932.26 | 0.03 | 0.05 | 0.60 | 0.32 |
| **FONT** | November | 11333.36 | 0.03 | 0.05 | 0.61 | 0.31 |

# References

Barbosa, A.M., R. Real and J.M. Vargas. 2009. “Transferability of environmental favourability models in geographic space: the case of the Iberian desman (*Galemys pyrenaicus*) in Portugal and Spain.” *Ecological modelling* 220(5):747–754.

Bridge, T.C.L., Z. Huang, R. Przeslawski, M. Tran, J. Siwabessy, K. Picard, A.E. Reside, M. Logan, S.L. Nichol and M.J. Caley. 2020. “Transferable, predictive models of benthic communities informs marine spatial planning in a remote and data-poor region.” *Conservation Science and Practice* 2(9):e251.

Cognat, M., F. Ganthy, I. Auby, F. Barraquand, L. Rigouin and A. Sottolichio. 2018. “Environmental factors controlling biomass development of seagrass meadows of Zostera noltei after a drastic decline (Arcachon Bay, France).” *Journal of Sea Research* 140:87–104.

Fix, E. and J.L. Hodges. 1989. “Discriminatory analysis. Nonparametric discrimination: Consistency properties.” *International Statistical Review/Revue Internationale de Statistique* 57(3):238–247.

Heikkinen, R.K., M. Marmion and M. Luoto. 2012. “Does the interpolation accuracy of species distribution models come at the expense of transferability?” *Ecography* 35(3):276–288.

Johnson, S. and K. Mengersen. 2012. “Integrated Bayesian network framework for modeling complex ecological issues.” *Integrated environmental assessment and management* 8(3):480–490.

Lauria, V., A.M. Power, C. Lordan, A. Weetman and M.P. Johnson. 2015. “Spatial transferability of habitat suitability models of Nephrops norvegicus among fished areas in the Northeast Atlantic: sufficiently stable for marine resource conservation?” *PLoS One* 10(2):e0117006.

Moon, J.B., T.H. Dewitt, M.N. Errend, R.J.F. Bruins, M.E. Kentula, S.J. Chamberlain, M.S. Fennessy and K.J. Naithani. 2017. “Model application niche analysis: assessing the transferability and generalizability of ecological models.” *Ecosphere* 8(10):e01974.

Mor´an-Ord´on˜ez, A., J.J. Lahoz-Monfort, J. Elith and B.A. Wintle. 2017. “Evaluating 318 continental-scale species distribution models over a 60-year prediction horizon: what factors influence the reliability of predictions?” *Global Ecology and Biogeography* 26(3):371–384.

Moreno-Amat, E., R.G. Mateo, D. Nieto-Lugilde, N. Morueta-Holme, J.-C. Svenning and I. Garc´ıa-Amorena. 2015. “Impact of model complexity on cross-temporal transferability in Maxent species distribution models: An assessment using paleobotanical data.” *Ecological Modelling* 312:308–317.

Peterson, A., M. Papes and M. Eaton. 2007. “Transferability and model evaluation in ecological niche modeling: a comparison of GARP and Maxent.” *Ecography* 30(4):550–560.

Randin, C.F., T. Dirnb¨ock, S. Dullinger, N.E. Zimmermann, M. Zappa and A. Guisan. 2006. “Are nichebased species distribution models transferable in space?” *Journal of biogeography* 33(10):1689–1703.

Rapacciuolo, G., D.B. Roy, S. Gillings, R. Fox, K. Walker and A. Purvis. 2012. “Climatic associations of British species distributions show good transferability in time but low predictive accuracy for range change.” *PLoS One* 7(7):e40212.

Real, R., A.M. Barbosa and J.M. Vargas. 2006. “Obtaining environmental favourability functions from logistic regression.” *Environmental and Ecological Statistics* 13(2):237–245.

Sequeira, A.M.M., C. Mellin, H.M. Lozano-Montes, M.A. Vanderklift, R.C. Babcock, M.D.E. Haywood, J.J. Meeuwig and M.J. Caley. 2016. “Transferability of predictive models of coral reef fish species richness.” *Journal of Applied Ecology* 53(1):64–72.

Sequeira, A.M.M., P.J. Bouchet, K.L. Yates, K. Mengersen and M.J. Caley. 2018. “Transferring biodiversity models for conservation: opportunities and challenges.” *Methods in Ecology and Evolution* 9(5):1250–1264.

Strauss, B. and R. Biedermann. 2007. “Evaluating temporal and spatial generality: How valid are species– habitat relationship models?” *ecological modelling* 204(1-2):104–114.

Tuanmu, M.-N., A. Vina, G.J. Roloff, W. Liu, Z. Ouyang, H. Zhang and J. Liu. 2011. “Temporal transferability of wildlife habitat models: implications for habitat monitoring.” *Journal of Biogeography* 38(8):1510– 1523.

Vanreusel, W., D. Maes and H. Van Dyck. 2007. “Transferability of species distribution models: a functional habitat approach for two regionally threatened butterflies.” *Conservation biology* 21(1):201–212.

Wang, X., D.J. Nott, C.C. Drovandi, K. Mengersen and M. Evans. 2018. “Using history matching for prior choice.” *Technometrics* 60(4):445–460.

Wenger, S.J. and J.D. Olden. 2012. “Assessing transferability of ecological models: an underappreciated aspect of statistical validation.” *Methods in Ecology and Evolution* 3(2):260–267.

Wu, P.P.-Y., K. Mengersen, K. McMahon, G.A. Kendrick, K. Chartrand, P.H. York, M.A. Rasheed and M.J. Caley. 2017. “Timing anthropogenic stressors to mitigate their impact on marine ecosystem resilience.” *Nature communications* 8(1):1–11.
